# Genetic analysis of the human infective trypanosome *Trypanosoma brucei gambiense*: chromosomal segregation, crossing over, and the construction of a genetic map

**DOI:** 10.1186/gb-2008-9-6-r103

**Published:** 2008-06-22

**Authors:** Anneli Cooper, Andy Tait, Lindsay Sweeney, Alison Tweedie, Liam Morrison, C Michael R Turner, Annette MacLeod

**Affiliations:** 1 Wellcome Centre for Molecular Parasitology, Glasgow Biomedical Research Centre, University Place, Glasgow, G12 8TA, UK; 2 Division of Infection and Immunity, Faculty of Biomedical and Life Sciences, Glasgow Biomedical Research Centre, University Place, Glasgow, G12 8TA, UK

## Abstract

**Background:**

*Trypanosoma brucei *is the causative agent of human sleeping sickness and animal trypanosomiasis in sub-Saharan Africa, and it has been subdivided into three subspecies: *Trypanosoma brucei gambiense *and *Trypanosoma brucei rhodesiense*, which cause sleeping sickness in humans, and the nonhuman infective *Trypanosoma brucei brucei*. *T. b. gambiense *is the most clinically relevant subspecies, being responsible for more than 90% of all trypanosomal disease in humans. The genome sequence is now available, and a Mendelian genetic system has been demonstrated in *T. brucei*, facilitating genetic analysis in this diploid protozoan parasite. As an essential step toward identifying loci that determine important traits in the human-infective subspecies, we report the construction of a high-resolution genetic map of the STIB 386 strain of *T. b. gambiense*.

**Results:**

The genetic map was determined using 119 microsatellite markers assigned to the 11 megabase chromosomes. The total genetic map length of the linkage groups was 733.1 cM, covering a physical distance of 17.9 megabases with an average map unit size of 24 kilobases/cM. Forty-seven markers in this map were also used in a genetic map of the nonhuman infective *T. b. brucei *subspecies, permitting comparison of the two maps and showing that synteny is conserved between the two subspecies.

**Conclusion:**

The genetic linkage map presented here is the first available for the human-infective trypanosome *T. b. gambiense*. In combination with the genome sequence, this opens up the possibility of using genetic analysis to identify the loci responsible for *T. b. gambiense *specific traits such as human infectivity as well as comparative studies of parasite field populations.

## Abstract 2

A high-resolution genetic linkage map of the STIB 386 strain of *Trypanosoma brucei gambiense* is presented.

## Background

Genetic maps can be used to establish the order, location, and relative distance of genetic markers in organisms that undergo sexual recombination, as well as to define some of the basic features of recombination. Their most important application, however, is in the identification of loci that determine traits or phenotypes that differ between individuals by linkage analysis. The importance of the genetic mapping of traits as a tool, coupled with positional cloning, is particularly high when analyzing both simple and complex phenotypes for which there are no obvious candidate genes, and it provides a complementary tool with which to reverse genetics in order to analyze gene function.

Genetic maps have been generated for a number of haploid eukaryotic pathogens including *Plasmodium falciparum *[1], *Plasmodium chabaudi chabaudi *[2], *Toxoplasma gondii *[3], and *Eimeria tenella *[4]. The genetic linkage approach, using such maps, has been an important tool for mapping genes which are responsible for drug resistance [5,6], virulence [7-10], and strain specific immunity [11]. An important feature of the maps of all these organisms is that the physical size of the recombination unit is relatively small, ranging from 17 kilobases (kb) per cM in the case of *P. falciparum *[1] to 100 to 215 kb in the case of *E. tenella *and *T. gondii *[3,4,12]. This means that the analysis of relatively few progeny can provide high mapping resolution; this is in contrast to higher eukaryotes, in which the physical size of the recombination unit is usually considerably greater [13].

The use of this approach to identify loci linked to traits of interest in diploid pathogens has been more limited. This is either because there is no evidence for a system of genetic exchange (a crucial requirement for the application of this approach) or the basic rules of how genetic exchange occurs have not been fully defined. *Trypanosoma brucei *is a diploid protozoan parasite for which genetic exchange has successful been demonstrated, first by Jenni and coworkers [14] and in multiple crosses since [15]. This tsetse-transmitted parasite is the causative agent of human sleeping sickness and animal trypanosomiasis in sub-Saharan Africa, and can be subdivided into three morphologically identical subspecies: *Trypanosoma brucei gambiense *and *Trypanosoma brucei rhodesiense*, which are the cause of sleeping sickness in humans; and the nonhuman infective *Trypanosoma brucei brucei *subspecies.

Over the past 20 years, several experimental genetic crosses have been performed both between and within subspecies (for review [15]). This includes the crossing of two *T. b. brucei *and a *T. b. gambiense *strain in all pair-wise combinations [16], from which the products of mating have been defined as the equivalent of F_1 _progeny, with the inheritance of alleles at parental heterozygous loci conforming to Mendelian ratios [17]. The strains used in these crosses (STIB 247, STIB 386, and TREU 927) were isolated from different regions of Africa and different hosts. They also differ in a range of phenotypes [18], allowing the genetic basis of these differences to be analyzed.

The chromosomes of *T. brucei *do not condense during mitosis, but the nuclear karyotype has been observed by separating chromosomes using pulsed field gel electrophoresis (PFGE) [19]. Unusually, the genome consists of three classes of chromosomes, which are categorized by size based on their migration in an electric field. The 11 diploid megabase chromosomes (1 to 6 megabases [Mb]) contain the housekeeping genes [20,21]; one to seven intermediate chromosomes (200 to 900 kb) of uncertain ploidy contain expression sites for the variant surface glycoprotein (VSG) genes, which are involved in antigenic variation [22]; and approximately 100 transcriptionally silent minichromosomes (50 to 150 kb) contain sequences for expanding the repertoire of available VSG genes [23,24].

A project to sequence the megabase chromosomes of *T. brucei *has resulted in the availability of the genome sequence for one of the *T. b. brucei *isolates, namely TREU 927 [25], which has been used in several of the genetic crosses, and this has been utilized by our laboratory to generate a genetic map for this strain [26]. It is the *T. b. gambiense *subspecies, however, that is responsible for the majority of current human African trypanosomiasis infections in sub-Saharan Africa [27,28]. Although it is related to *T. b. brucei*, it differs in several important phenotypic characteristics, such as human infectivity. A separate *T. b. gambiense *genetic map is therefore desirable for the study of specific mechanisms of disease in this pathogenic subspecies.

For this reason, the strain STIB 386 is of particular interest as it was isolated from a human in West Africa and is consequently defined as *T. b. gambiense*. Two types of this human-infective subspecies have been identified, types 1 and 2 [29], that differ in biologic features such as growth in rodents and constitutive or nonconstitutive expression of resistance to lysis by human serum (a measure of human infectivity); they also differ at the molecular level, based on findings with a range of polymorphic markers [30,31].

The STIB 386 strain is a type 2 *T. b. gambiense*, with the characteristics of ready growth in rodents and variable expression of human serum resistance [32] as well as differing in a number of other phenotypes from strain STIB 247. We have previously reported data from a cross between these two strains (STIB 386 × STIB 247) and the Mendelian segregation of 11 markers, each on separate chromosomes, into 38 independent F_1 _progeny isolated from the cross [17]. As an essential and important step toward using this cross to map genes determining traits of importance in the human-infective subspecies of *T. brucei*, we report the construction of a genetic map of the STIB 386 strain of *T. b. gambiense*, defining the key features of recombination and providing a comparative analysis with the genetic map of *T. b. brucei *strain TREU 927.

## Results

### Identification of heterozygous markers and the genotyping of F_1 _progeny

The *T. brucei *genome sequence from strain TREU 927 had previously been screened using the Tandem Repeat Finder program [33] to identify microsatellites, which were evenly distributed across the genome. A total of 810 pairs of primers was designed to the unique sequence flanking each microsatellite locus [26]. These primers were used to amplify by PCR the microsatellites from the two parental stocks, STIB 386 and STIB 247, thus identifying markers that were heterozygous and could therefore be used to construct a genetic map of STIB 386. Heterozygous markers were defined by the amplification of two different sized PCR products in STIB 386, which could be easily separated and visualized by gel electrophoresis.

In all, 99 potentially informative markers were identified using this method and so could be used for the construction of a partial genetic map, whereas the remaining 711 markers either amplified a homozygous band in STIB 386 or failed to amplify any PCR product. Of these 99 heterozygous markers, 47 had also previously been found to be heterozygous for TREU 927 and so were included in the construction of both the *T. b. brucei *and *T. b. gambiense *genetic maps.

Following this initial microsatellite screen, further markers were sought to fill in regions of the genome that were not covered by a heterozygous marker for STIB 386. An additional 215 primer pairs were designed to screen further microsatellites from these regions, resulting in the identification of an additional 20 heterozygous markers and a total marker coverage of 119 heterozygous markers. Overall the level of heterozygosity for all the markers screened is significantly lower, at 12.5%, than the value of 20% reported for the genome strain (χ^2 ^[1 degree of freedom] = 27.3; *P *< 0.01) [26]. Thirty-eight F_1 _progeny clones from the cross between STIB 386 and STIB 247 were genotyped with the 119 markers and the segregation patterns in the progeny were scored to generate a full genotype of each progeny clone (Additional data file 1 contains the complete segregation data).

### Construction of the STIB 386 genetic linkage map

**Figure 1:**
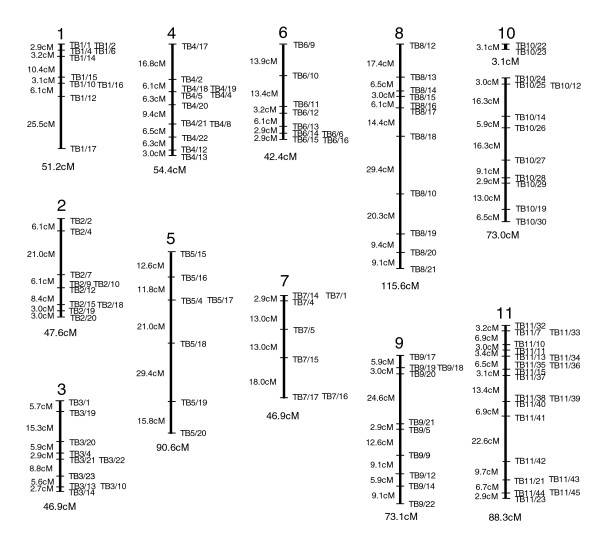
Genetic linkage maps corresponding to the 11 Mb chromosomes of *Trypanosoma brucei gambiense*. Every microsatellite marker (shown to the right of each linkage group) has been anchored to the physical map, and the physical location (derived from the *T. b. brucei *genome sequence [25]) is identified in the supplementary data (Additional data file 1). The corresponding genetic distances between intervals is shown in cM on the left of each map and the total genetic size of each linkage group given below.

The inheritance pattern of STIB 386 alleles, at each heterozygous locus, in the 38 F_1 _progeny was determined (Additional data file 1) and the segregation data used to construct a genetic map using the Map Manager QTX program [34]. This linked the 119 markers into 12 linkage groups, which correspond to the 11 housekeeping chromosomes. The genetic linkage map of each chromosome is shown in Figure 1, and although ten chromosomes (1, 2, 3, 4, 5, 6, 7, 8, 9, and 11) consist of one linkage group each, chromosome 10 currently comprises two groups. The main characteristics of the linkage groups obtained are summarized in Table 1. The genetic distances, based on the number of recombination units between each marker, are expressed in centiMorgans, which added together for all 12 linkage groups gave a total genetic map length of 733.1 cM. The size of each chromosome and the physical distances between markers were based on the TREU 927 *T. b. brucei *sequence [25]. Using these figures, the genetic map covers 17.9 Mb, which equates to an approximate genome coverage of 70%. However, this calculation includes the gene-poor subtelomeric regions, which the genetic map does not extend into because of the difficulties in identifying unique sequences in these regions.

**Table 1:** Characteristics of the genetic linkage maps of *Trypanosoma brucei gambiense*

Chromosome	Number of markers	Genetic length (cM)^a^	Physical size (Mb)^b^	Recombination Frequency (kb/cM)	Average number of crossover events/meiosis
1	10	51.20	0.74	14.53	0.46
2	10	47.60	0.74	15.46	0.42
3	10	46.90	1.25	26.74	0.42
4	12	54.40	1.05	19.30	0.50
5	7	90.60	1.20	13.29	0.74
6	9	42.40	0.94	22.13	0.35
7	7	46.90	1.65	35.08	0.40
8	11	115.60	2.30	19.88	0.95
9	10	73.10	2.10	28.67	0.65
10^c^	12	76.10	2.50	32.85	1.08
11	21	88.30	3.42	38.76	0.71

Average				24.40	0.61

Total	119	733.10	17.89		

^a^Total genetic length was calculated by the addition of recombination units between each marker. ^b^Physical distances were calculated from the *T. b. brucei *genome sequence [25]. ^c^Chromosome 10 is a combination of two linkage groups.

On average, the crossover frequency was found to be 0.6 crossovers/chromosome/individual progeny clone in the mapped population (Table 1) and the average recombination unit size is 24.4 kb/cM. This provides a 9 cM resolution genetic map with a 90% probability of mapping any locus to within 11 cM (268 kb). The physical position of each microsatellite marker, based on the genome sequence of *T. b. brucei *[25], allows us to compare the position of markers in the physical map of *T. b. brucei *and the genetic map of *T. b. gambiense*, revealing that synteny is conserved for all markers on all chromosomes (Additional data files 1 and 2).

### Marker segregation proportions

**Figure 2:**
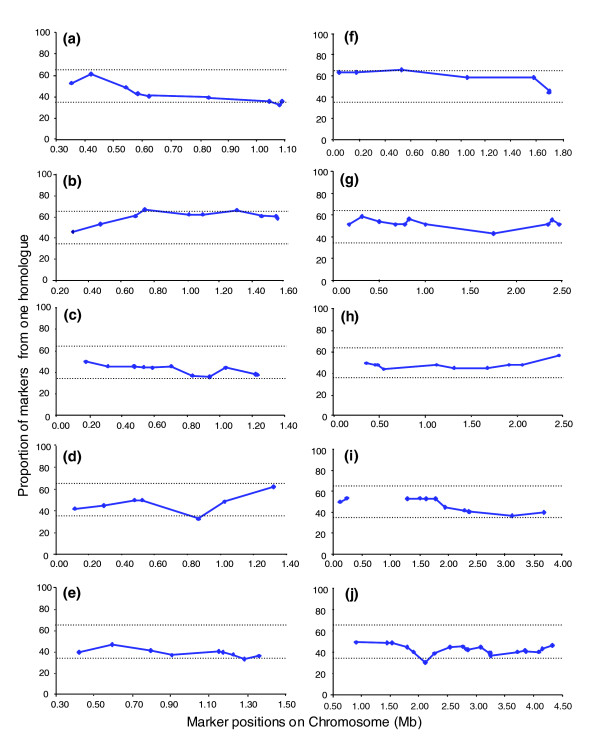
Genotype segregation proportions. Genotype segregation proportions for all microsatellite markers present on chromosomes: **(a) **2, **(b) **3, **(c) **4, **(d) **5, **(e) **6, **(f) **7, **(g) **8, **(h) **9, **(i) **10, and **(j) **11. Dashed horizontal lines indicate the approximate 95% probability range for equal segregation of alleles.

The availability of segregation data across the length of each chromosome allows a full analysis of the inheritance of the STIB 386 parental chromosome homologs. The ratio of segregation of alleles for each heterozygous marker was calculated along each chromosome with the 95% confidence limits of a 1:1 segregation with 38 F_1 _progeny. This analysis had previously been conducted for the STIB 386 map of one of the smallest chromosomes, namely chromosome 1, and detected a region of significant distortion across the left arm of the chromosome [17]. Segregation analysis has now been performed on the remaining ten chromosomes (Figure 2) and this shows no evidence of distortion from a 1:1 segregation ratio across the length of chromosomes 4, 8, 9, or 10. On chromosomes 2, 5, 6, 7, and 11 there is one marker per chromosome, and on chromosome 3 there are two markers that have been inherited at proportions just outside the 95% confidence limits. However, it should be considered that this totals only seven out of 109 markers analyzed (6%), which is close to the 5% of outliers that would be expected with 95% confidence intervals and thus are unlikely to signify regions of true segregation distortion. Therefore, the previously reported region of chromosome 1 remains the only region of the STIB 386 genetic map for which there is evidence of any significant segregation distortion. The origin of this distortion is not known, but one possibility is that it is the result of postmeiotic selection acting on the uncloned progeny during growth in mice before isolation.

### Variation in recombination between chromosomes

**Figure 3:**
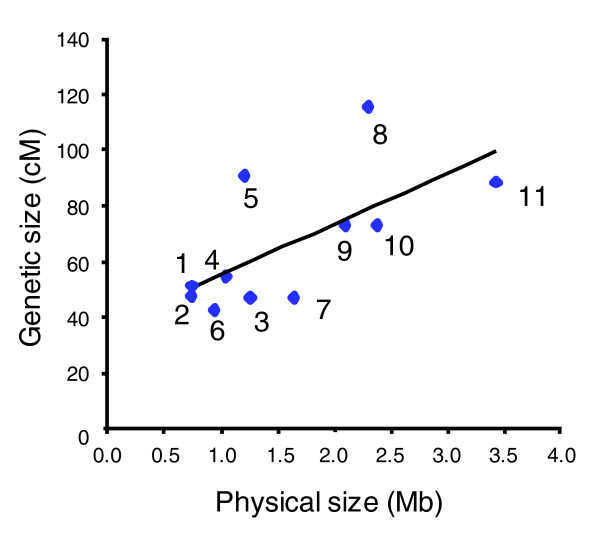
The genetic size of each linkage group relative to its physical size. A comparison of the total genetic size of each linkage group against the predicted physical distance, calculated from the *T. b. brucei *genome sequence [25]. The line shown was determined by linear least squares regression analysis.

**Figure 4:**
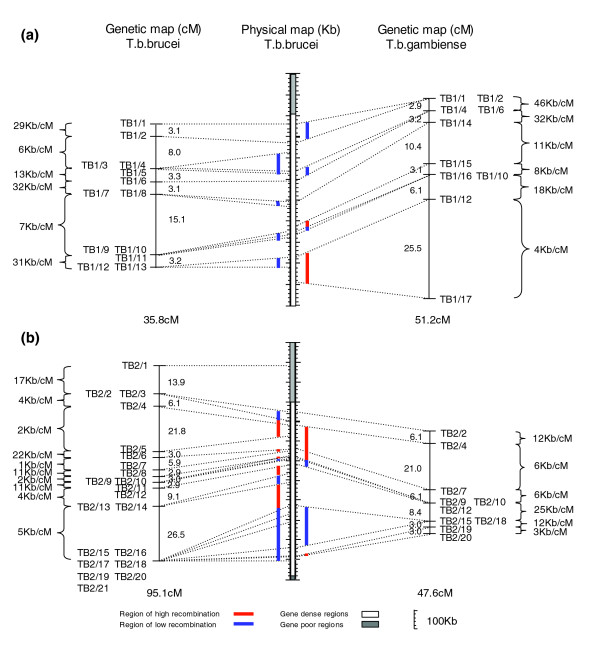
Comparison with the physical and genetic maps of *Trypanosoma brucei brucei*. The genetic maps of *T. b. brucei *isolate TREU 927 and *T. b. gambiense *isolate STIB 386 are shown alongside the TREU 927 physical map of the same chromosome for **(a) **chromosome 1 and **(b) **chromosome 2. The average physical size of a recombination unit between each marker is given in kb/cM and the genetic distance given in cM. Dashed lines link the position of all markers on the physical map to their relative position on the genetic maps. Hot and cold spots are defined as threefold more or less recombination than average for each genetic map and indicated against the physical map by red and blue bars, respectively.

Although the average rate of recombination in the *T. b. gambiense *map was found to be 24.4 kb/cM, there is variation both between and within the chromosomes, as is common in many other eukaryotic organisms [35]. A correlation of the physical and genetic sizes of every chromosome in the map is shown in Figure 3, and the average physical size of a recombination unit ranges from a high of 39 kb/cM on chromosome 11 to a low of 13 kb/cM on chromosome 5 (Table 1). Variation is also evident between specific intervals across chromosomes where a map unit can vary from under 1 kb/cM up to 170 kb/cM on the same chromosome (chromosome 11; Additional data file 2) representing extremes in recombination frequency. If we define hot and cold spots of recombination as three times less (cold) or three times more (hot) than the average recombination rate, the boundaries for defining hot and cold regions can be set at under 8 kb/cM and over 73 kb/cM, respectively, based on an average physical size of a recombination unit of 24 kb/cM. Analysis of crossovers in the STIB 386 × STIB 247 progeny revealed that variation in recombination frequency between markers is common, producing a least one hot or cold region on every chromosomes and a total of 15 hot and 27 cold spots overall (Figure 4 and Additional data file 2).

Variation in recombination was also noted as a common feature in the *T. b. brucei *TREU 927 map [26]. Data from the *T. b. brucei *genetic map was re-analyzed alongside the *T. b. gambiense *map to identify regions of high and low recombination using the same definition of boundaries. Based on an average physical recombination unit size of 15.6 kb/cM for TREU 927, hot and cold spot boundaries could therefore be defined as under 5.2 kb/cM and over 46.8 kb/cM, respectively. As a result of this analysis, a similar number of hot and cold regions were identified on the TREU 927 map, with a total of 20 hot and 32 cold spots overall (Figure 4 and Additional data file 2).

A more detailed comparison of these regions with those identified on STIB 386 was then performed, and four areas of high recombination (hot) and ten of low recombination (cold) were found to overlap the same physical location on both genetic maps. Chromosome 2, for example (Figure 4b), has a region of higher recombination toward the center of the chromosome (denoted in red), which contains two of the STIB 386 hot spots and four of the TREU 927 hot spots, as well as a large shared cold spot (denoted in blue) toward the end of the chromosome, with no evidence of recombination over a distance of more than 200 kb on either map. In contrast, there are also several regions, where a STIB 386 hot spot corresponds to a cold spot on TREU 927, as illustrated at the end of chromosome 1 (Figure 4a) and *vice versa *(for example, chromosome 8; Additional data file 2). Although local variation in crossover frequency appears to be a common feature of both the *T. b. brucei *and *T. b. gambiense *maps, this balances out over the full length of each chromosome, with the net result being that the total genetic distance of linkage groups is correlated with their physical size (Figure 3).

### Comparison of the genetic maps of *T. b. gambiense *and *T. b. brucei *and the physical map of *T. b. brucei*

The linkage groups of the STIB 386 genetic map comprise a total genetic distance of 733.1 cM covering a physical distance of 17.9 Mb, compared to a genetic map of 1,157 cM covering 18.06 Mb for the *T. b. brucei *TREU 927 map [26]. Although the genetic distance covered by the STIB 386 map is smaller, there is no significant difference in frequency of recombination (kb/cM) between the two subspecies (χ^2 ^[1 degree of freedom] = 1.936; *P *= 0.164), and they contain very similar marker densities (average cMs between intervals) of 9.0 cM for STIB 386 and 9.5 cM for TREU 927.

Because 47 markers are informative in both the *T. b. brucei *and *T. b. gambiense *maps, this allows a direct evaluation of genetic distances between the maps, and comparison with the physical *T. b. brucei *map. For six chromosomes for which there are four or more shared markers (chromosomes 1, 2, 3, 4, 9 and 11), synteny in terms of marker order is conserved (Figure 4 and Additional data file 2). The rest of the chromosomes have fewer shared markers, making comparisons less informative, but no inconsistencies between the genetic map and the physical map of TREU 927 were detected. The karyotype of both strains has been determined by PFGE [20] and, in terms of chromosome size, seven of the chromosome pairs of STIB 386 are found to be considerably larger than those of TREU 927 (chromosomes 1, 4, 6, 7, 8, 9, and 10). If these physical size differences occurred in regions of each chromosome covered by the genetic map, then one would predict that the recombination frequency of the STIB 386 chromosomes would be correspondingly higher and result in larger genetic distances between markers, but this does not appear to be the case.

To illustrate the similarities and differences between chromosomes, the data for chromosomes 1 and 2 are illustrated (Figure 4). For chromosome 2, the physical size of the chromosome is similar in both isolates based on PFGE [20], but the size of the genetic maps differ significantly. Comparing only the region of the chromosome represented by both genetic maps, from marker TB2/2 to TB2/20, the genetic distances for *T. b. brucei *and *T. b. gambiense *are 81.2 cM and 47.6 cM, respectively (Figure 4b), which is significantly different (χ^2 ^[1 degree of freedom] = 8.765; *P *< 0.01). The difference in genetic distance between the chromosome two maps is largely due to a hotspot of recombination in the interval between markers TB2/20 and TB2/12 in *T. b. brucei *(35.6 cM), which in not present in *T. b. gambiense *(14.4 cM) at the same marker interval. However, for chromosome 1 (Figure 4a), comparing the distance represented by the two genetic maps (35.8 cM and 25.1 cM), the difference is not significant (χ^2 ^[1 degree of freedom] = 1.88; *P *= 0.17), despite the physical size of chromosome 1 in the *T. b. gambiense *strain STIB 386 being estimated to be almost twice that of TREU 927 [20].

### Mutation frequency

A single spontaneous mutation event, generating a novel sized allele product, distinct from the parental alleles, was detected when genotyping the progeny clones. This mutation occurred at marker TB6/15, resulting in a mutation frequency at this locus of 0.028 mutants/alleles genotyped. Combined with all other markers this produces an overall mutation frequency of 0.00024 mutants/alleles genotyped, which is consistent with the mutation frequency of 0.0003 mutants/alleles genotyped reported for the *T. b. brucei *strain TREU 927 [26]. In contrast to the TREU 927 mutant loci, the allele in question had lost repeats resulting in an allele smaller than either of the parental alleles. The origin of the mutation has not been determined, but as the original parental allele is not detected in addition to the mutant, the mutation is unlikely to have arisen during vegetative growth of the progeny clone, but before the cloning process, probably at meiosis.

## Discussion

Genetic linkage maps have been determined for a number of parasites, including the haploid apicomplexa species *Plasmodium falciparum *[1], *Plasmodium chabaudi chabaudi *[2], *Eimeria tenella *[4], and *Toxoplasma gondii *[3], and recently the first map for the diploid trypanosomatid *T. b. brucei *was reported [26]. Here, we advance knowledge of this parasite by reporting the construction of the first linkage map of a human-infective strain of the *T. b. gambiense *subspecies to provide a basis for expanding studies on important biological traits in this line such as human infectivity and virulence.

The average recombination rate in this genetic map (24.4 kb/cM) is close to the values reported for *T. b. brucei *[26], *P. falciparum *[1], and other organisms with a similar size genome [13]. However, as observed for a variety of other eukaryotes, there is considerable variation in the physical size of a cM. Similar hot and cold spots of meiotic recombination have been reported for a wide variety of eukaryotic species [35] and were also identified on the *T. b. brucei *TREU 927 map [26,36,37]. Although local variation in crossover frequency appears to be a common feature of both the *T. b. brucei *and *T. b. gambiense *maps, this balances out over the full length of each chromosome, with the total genetic distance of chromosomes correlated with their physical sizes for the *T. b. brucei *map [26] and to a lesser degree with the *T. b. gambiense *map, with the caveat that the sequence data of *T. b. brucei *was used to as a basis for estimating the physical size for *T. b. gambiense*.

Size polymorphism in the megabase chromosomes of *T. brucei *has been documented both between isolates and between homologs within a single parasite genome [21,38]. PFGE resolution of the molecular karyotype for the genetic map isolate STIB 386 showed that at least seven out of 11 chromosome pairs were larger in size than those in the *T. b. brucei *genome reference strain TREU 927 [20]. On this basis we might therefore anticipate the genetic size of these chromosomes to reflect this physical size difference, with larger genetic distances in those chromosomes that are larger in the *T. b. gambiense *subspecies. Interestingly, though, we found no significant difference in recombination, measured in terms of average map unit size, between the two strains. Indeed, where distance between markers present on both genetic maps were examined, STIB 386 was frequently found to have the smaller genetic map distance, despite the predicted size of homologs being up to twice that of TREU 927 [20,21].

Considerable chromosome size variation between isolates has been reported in many protozoan parasites with little or no effect on gene content. Variations in chromosome size between strains of 10-50% in *Plasmodium falciparum *[39-41], *Leishmania *spp. [42-44], and *Trypanosoma cruzi *[45] have been attributed primarily to changes in repeat regions in the subtelomeric sequence. This polymorphism is even more extreme in *T. brucei *isolates, in which chromosome plasticity results in homologs varying up to fourfold between isolates [46] and even twofold within a single genome [20,21,46], without an apparent loss of linkage in coding regions.

Comparisons of the Trypanosomatid genome sequence data, comprising the *T. brucei*, *T. cruzi *and *Leishmania major *species, has uncovered a common chromosomal arrangement with a central core exhibiting extensive synteny [47]. Within *T. brucei *isolates, comparative studies of homologous chromosomes have as yet failed to identify any associated loss of synteny or translocation in coding regions, even between very size divergence chromosomes. In one such study, DNA microarray analysis of the genome content variation of chromosome 1, one of the most size variable chromosomes, was used to identify regions of copy number polymorphism between strains [48]. As observed with related protozoan pathogens, the majority of the extensive size variation between isolates appeared to be concentrated in the subtelomerically located genes, including the VSGs, VSG expression site associated genes, and highly polymorphic gene families such as the retrotransposon hot spot and leucine-rich repeat protein genes. Variation in copy number of these repeat elements was found to compose as much as 75% of the length of a homolog. In contrast, 90% of the diploid core showed little evidence of significant copy number variation, with polymorphisms mainly limited to tandemly repeated gene arrays such as tubulin, histone H3, and the pteridine transporters.

Our comparison of the *T. b. brucei *strain TREU 927 and *T. b. gambiense *strain STIB 386 genetic maps is in agreement with these findings. We report no inconsistency in the marker order or average map unit size between the STIB 386 genetic map and that of *T. b. brucei*. Some strain-specific local variation in the recombination rate between shared markers pairs were identified, which may be attributed to local physical size differences or variation in tandemly repeated gene arrays within the coding regions. Overall, though, our data appear to be in agreement with a conservation of synteny between the two subspecies, with the majority of the variation accounting for chromosome size difference between the two strains focused outside the gene-rich coding region (in the sub-telomeres) and therefore not covered by the genetic map.

The genetic distances in the map reflect the number of recombination events that have occurred in the population during meiosis. At least one reciprocal crossover per chromosome is considered essential for the successful disjunction of homologous chromosomes during meiosis [49]. It is therefore surprising that 48% of all STIB 386 chromosomes analyzed in this cross failed to exhibit evidence of any recombination events (a full analysis of crossovers in the progeny is available in Additional data file 3). Progeny averaged only 0.6 crossovers/chromosome compared with the 1.02 calculated for the TREU 927 map, despite comparable coverage of the genome. Indeed, in several progeny clones, evidence of recombination was extremely rare or, in the case of hybrid F492/50 bscl 23, entirely absent on all 11 chromosomes. The reasons for this low crossover frequency are unknown but may also be a consequence of the larger predicted genome size of the STIB 386 strain. Physical estimates of marker locations were established from the available TREU 927 sequence to produce a total predicted coverage of the genome of 70%. However, if the larger physical size of STIB 386 was due to extended subtelomeric regions, then this would leave an increased percentage of the genome outside of the gene-dense center, uncovered by the map. If the obligate crossover necessary to ensure faithful meiotic segregation of chromosomes is occurring outside the central core on some STIB 386 chromosomes and toward the subtelomeric regions at the ends of chromosomes, then it would not be detected by our analysis.

Estimations of the frequency at which spontaneous microsatellite mutations occur may enhance our understanding of the evolution and stability of such markers and their usefulness in genetic analysis of *T. brucei *populations. Few such estimates exist for *T. brucei*, but an approximate mutation rate of 0.0003 mutants/allele genotyped was reported in the *T. b. brucei *genetic map from the identification of two spontaneous mutation events in a dataset of 6,797 microsatellite alleles. In this *T. b. gambiense *genetic map the identification of a single spontaneous mutation event in a microsatellite marker appears to substantiate this (0.00024 mutants/allele genotyped). These estimates are based on only a small number of mutation events and thus can only be considered an approximation, but they are comparable to a similar mutation rate reported in the malaria parasite *Plasmodium falciparum *of 0.00016 mutants/allele genotyped [50]. Given that we have screened an additional 118 markers and found no mutations (about 4,500 events), we can be confident that the value we have obtained is a maximum. Although the screening of a significantly larger dataset of marker alleles would allow a more accurate mutation rate to be obtained, we consider that our high coverage of the genome sequence in the screen for informative microsatellite markers - coupled with the relatively low level of heterozygosity - make it unlikely we would find enough additional microsatellite markers from further screening to detect more mutations.

*T. b. gambiense *is related to *T. b. brucei*, but differs significantly in many phenotypic characteristics, most notably in their ability to infect humans. Indeed, the *T. b. gambiense *and *T. b. brucei *strains examined here not only differ in terms of human infectivity and pathogenesis, but also in their ability to establish midgut infections in the tsetse vector, to progress from the midgut to the salivary glands (transmission index), and in their ability to resist killing by a number of trypanocidal drugs used in the treatment of human African trypanosomiasis [18]. The availability of a genetic linkage map for *T. b. gambiense *opens up the possibility of identifying genes that determine these traits. The value of a genetic map for identifying loci that effectuate particular phenotypes is primarily determined by the recombination frequency of the organism, providing there is sufficient marker coverage of the genome. *T. brucei *has a relatively high crossover frequency compared with higher eukaryotes, which is comparable to that seen in *P. falciparum *[1] and 40 times higher than in humans [51]. With this recombination frequency the 9 cM resolution of this map will allow linkage of a phenotype to within 270 kb of a genomic locus with 90% probability. Once such linkage is identified, finer scale mapping would be warranted and, consequently, it may then be beneficial to isolate further progeny and increase the marker density to improve the resolution of the map in the specific area of the genome. Under these circumstances other genetic markers such as single nucleotide polymorphisms could be used to increase the density of markers within chromosomal regions of interest.

## Conclusion

The genome sequence of *T. b. brucei *was recently completed, and that for *T. b. gambiense *is underway. Although this has provided useful insights into gene function, there is still a large percentage of genes that have no known function or ortholog. Genetic mapping is a powerful tool, which can attribute functions to some of these genes. The power of this approach lies in the fact that it identifies genes involved in naturally occurring variation, requires no prior knowledge as to the nature of the genes involved in particular phenotypes, and it can identify genes involved in complex traits, which may be difficult to detect by other means. Such an approach has been validated in other parasites to identify genes involved in drug resistance in *Plasmodium falciparum *[52] and *Eimeria tenella *[4], and virulence in *Toxoplasma gondii *[3,7-9]. The genetic linkage map presented here is the first available for the human-infective trypanosome *T. b. gambiense*. In combination with the genome sequence, this opens up the possibility of using genetic analysis to identify the loci responsible for *T. b. gambiense *specific traits such as human infectivity.

## Materials and methods

### Origin of F_1 _progeny clones

The progeny clones from the cross between STIB 386 and STIB 247 used in the analysis and their derivation were described previously [16-18]. Briefly, tsetse flies were co-infected with a mixture of the two bloodstream stage parental trypanosomes and, after maturation within the flies to the metacyclic stage, the populations of trypanosomes from each fly were monitored for the presence of the products of mating. Once these were detected, cloned lines were established either by directly cloning metacyclic stage trypanosomes in individual immuno-suppressed mice or by cloning from bloodstream stage infections derived directly from feeding infected tsetse on a mouse. The resulting metacyclic and/or bloodstream, cloned populations from six mixed infected flies (F 8,19, 28, 29, 80 and 492) were then genotyped with two microsatellite markers JS2 [53] and PLC [26] and three minisatellites markers, MS42, CRAM, and 292 [54] that were heterozygous in one or both of the two parental stocks. This resulted in the identification of 38 independent F_1 _progeny clones from the cross, each of a different and unique genotype. A list of all hybrids and their genotypes is provided in the supplementary material (Additional data file 4).

### Preparation of DNA from trypanosomes

The parental stocks and the progeny clones derived from the cross were amplified in mice or by procyclic culture, and lysates of partially purified trypanosomes prepared as described previously [54].

### PCR amplification of mini and microsatellite markers

Primers were designed to the unique flanking sequences of tandemly repeated loci and used in PCR reactions, prepared in 10 μl reaction volumes containing the following: 45 mmol/l Tris-HCl (pH 8.8), 11 mmol/l (NH_4_)_2_SO_4_, 4.5 mmol/l MgCl_2_, 6.7 mmol/l 2-mercaptoethanol, 4.4 μmol/l EDTA, 113 μg/ml bovine serum albumin, 1 mmol/l each of the four deoxyribonucleotide triphosphates, 10 μmol/l each oligonucleotide primer, 0.5 units *Taq *DNA polymerase (Abgene, Epsom, UK), and 1 μl DNA template. Reactions were overlaid with mineral oil to prevent evaporation and amplification carried out in a Robocycler gradient 96 (Stratagene, La Jolla, CA, UK). All PCR reactions except the three minisatellites used for genotyping DNA stocks (CRAM, MS42 and 292) were amplified under the following conditions: 95°C for 50 seconds, 50°C for 50 seconds and 65°C for 50 seconds × 30 cycles. In the three minisatellites the following conditions were used: 95°C for 50 seconds, 60°C for 50 seconds and 65°C for 3 minutes × 30 cycles. PCR products were separated by gel electrophoresis on a 1% Seakem LE agarose gel for the 3 minisatellites and a 3% Nusieve GTG agarose gel for the microsatellites in 0.5 × TBE buffer containing 50 ng/ml ethidium bromide, visualized by UV illumination, and photographed for analysis.

### Identification of microsatellite markers and PCR screening

Primers for 810 markers, evenly distributed throughout the 11 chromosomes of the *T. brucei *genome, which had been designed for screening the TREU 927 × STIB 247 cross during construction of the TREU 927 *T. b. brucei *map, were available [26]. Primers for an additional 215 new markers were designed specifically for the construction of the STIB 386 map. Microsatellite markers were identified from the *T. brucei *genome sequence [25], accessed though the *Trypanosoma brucei *GeneDB resource [55] with the Tandem Repeat Finder program [56]. Candidate markers were identified as sequences containing more than ten copies of a repeat motif of two to six nucleotides with more than 70% sequence identity. Primer pairs were then designed for each microsatellite marker in the unique sequence flanking each repeat region using the PRIDE primer design program [57].

The primers were used to screen the parental STIB 386 and STIB 247 genomic DNA by PCR to identify loci that were heterozygous for allele size in STIB 386 and so would segregate in the progeny. These selected markers were PCR amplified from all 38 F_1 _progeny from the STIB 386 × STIB 247 cross and, following agarose gel electrophoresis, the inheritance of each STIB 386 parental allele in each progeny clone was determined for each microsatellite locus. All gels were independently scored by a second individual to ensure progeny genotypes were correctly assigned. The physical location of the markers on the *T. brucei *genome was determined by GeneDB BLASTN search of the primers against the *T. brucei *contigs database [55]. The details of the primers used and the markers scored are provided as supplementary material (Additional data file 1).

### Generation of a linkage map

A genetic map of STIB 386 was generated, based on the segregation of marker alleles in the F_1 _progeny, for loci heterozygous in the STIB 386 parent. The allele segregation data were analysed using the Map Manager QTX software [34], with a Haldane map function and the highest level of significance for linkage criteria, giving a probability of type 1 error *P *= 1 × e^-6^. Linkage between the adjacent physical markers was determined by a LOD (log of the odds) score of 5.5 or greater.

### Online resources

The genetic map, supplementary material, and additional information regarding how the genetic cross was performed is available on the Trypanosome Genetic Mapping Database website [58].

## Abbreviations

kb, kilobases; Mb, megabases; PCR, polymerase chain reaction; PFGE, pulsed field gel electrophoresis; VSG, variant surface glycoprotein.

## Authors' contributions

AC, ATa, MT and AML designed the experiments, analyzed the data, and wrote the manuscript. AC, LS, ATw, and LM carried out the experimental work. All authors read and approved the final manuscript.

## Additional data files

The following additional data are available with this paper. Additional data file 1 provides segregation data. Additional data file 2 provides a comparison with the physical and genetic maps of *T. b. brucei *for every chromosome. Additional data file 3 provides recombination data for every linkage group of every individual. Additional data file 4 provides the unique genotype pattern of each progeny clone.

## Supplementary Material

Additional data file 1 The segregation data for all the markers on each of the 11 Mb chromosomes is given. For each linkage group, markers are shown in map order, alongside: primer pair sequences, chromosomal location of the primers based on the available 927 sequence, estimated size of the PCR product, genotype of the STIB 386 parental line (AB), and inheritance pattern (either A or B) in the progeny clones for each marker. Novel sized alleles are marked as mutants. Click here for file

Additional data file 2 The genetic maps of *T. b. brucei *isolate TREU 927 and *T. b. gambiense *isolate STIB 386 are shown alongside the TREU 927 physical map of every chromosome. The average physical size of a recombination unit between each marker is shown on the outside of each map in kb/cM and the genetic distance, given in cM, shown on the inside. Dashed lines link the position of all markers on the physical map to their relative position on the genetic maps, based on the TREU 927 sequence. Hot and cold spots are defined here as threefold more or less recombination than average for each genetic map and indicated against the physical map by red and blue bars, respectively. Click here for file

Additional data file 3 A breakdown of the number of recombination events for every chromosome linkage group of every individual is given and the total and average for each individual and linkage group calculated. Click here for file

Additional data file 4 The name and relevant genotypes of the parental strains and 38 unique F1 progeny derived from the STIB 386 × STIB 247 crosses that were analysed for the construction of the *T. b. gambiense *linkage map. Inheritance of marker alleles from both parents for 2 microsatellites (JS2 and PLC) and 3 minisatellites (CRAM, 292 and MS42) were used as genotyping markers. Click here for file
