# Supplementary material for: Genetic analysis of the human infective trypanosome Trypanosoma brucei gambiense: chromosomal segregation, crossing over, and the construction of a genetic map
Source: Genome Biol. 2008 Jun 22;9(6):R103. doi: 10.1186/gb-2008-9-6-r103 (PMC2481433; doi:10.1186/gb-2008-9-6-r103)
Supplement: Additional data file 2 — The genetic maps of T. b. brucei isolate TREU 927 and T. b. gambiense isolate STIB 386 are shown alongside the TREU 927 physical map of every chromosome. The average physical size of a recombination unit between each marker is shown on the outside of each map in kb/cM and the genetic distance, given in cM, shown on the inside. Dashed lines link the position of all markers on the physical map to their relative position on the genetic maps, based on the TREU 927 sequence. Hot and cold spots are defined here as threefold more or less recombination than average for each genetic map and indicated against the physical map by red and blue bars, respectively. [file gb-2008-9-6-r103-S2.ppt]

## Slide 1
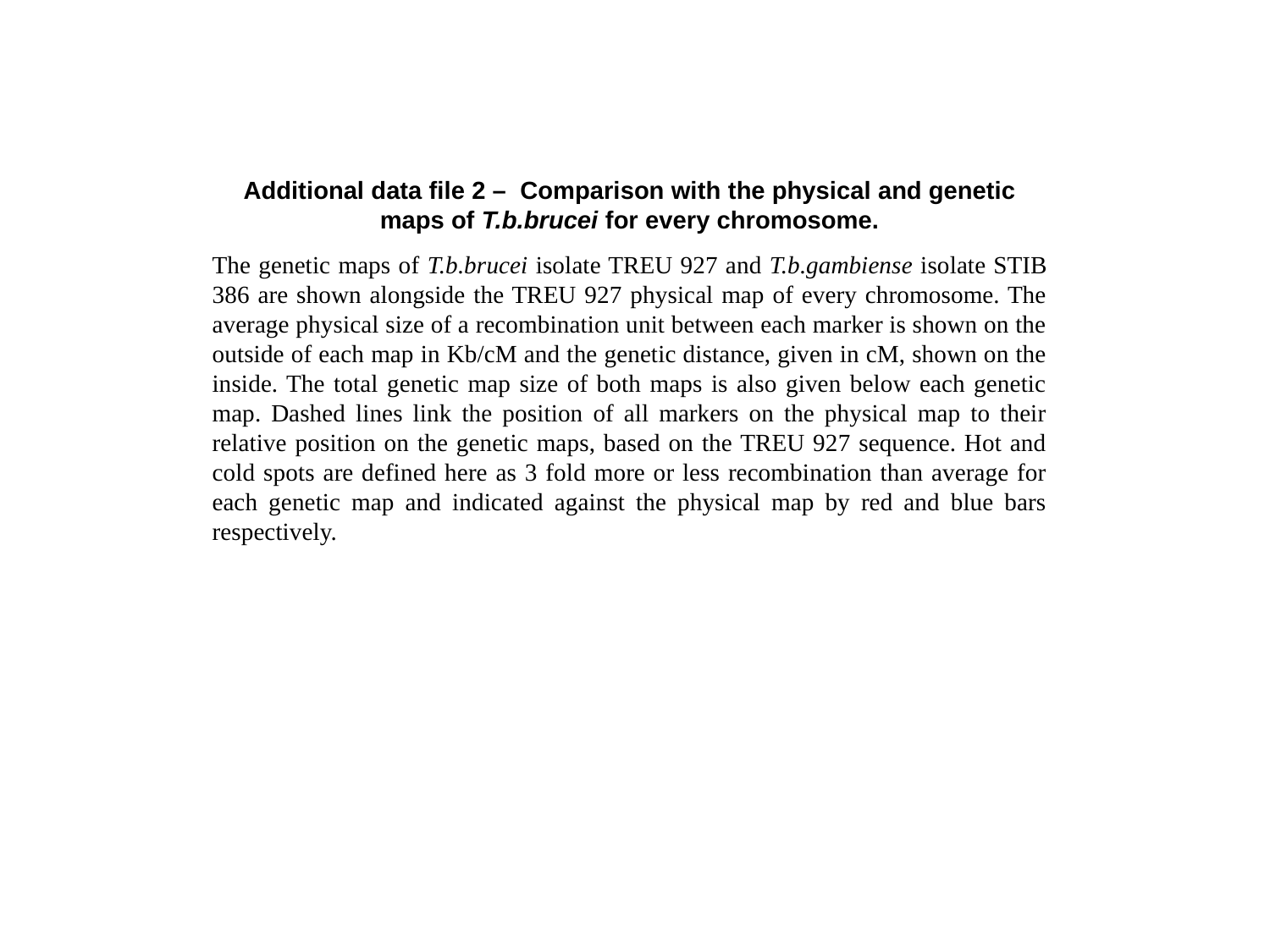

Additional data file 2 – Comparison with the physical and genetic maps of T.b.brucei for every chromosome.
The genetic maps of T.b.brucei isolate TREU 927 and T.b.gambiense isolate STIB 386 are shown alongside the TREU 927 physical map of every chromosome. The average physical size of a recombination unit between each marker is shown on the outside of each map in Kb/cM and the genetic distance, given in cM, shown on the inside. The total genetic map size of both maps is also given below each genetic map. Dashed lines link the position of all markers on the physical map to their relative position on the genetic maps, based on the TREU 927 sequence. Hot and cold spots are defined here as 3 fold more or less recombination than average for each genetic map and indicated against the physical map by red and blue bars respectively.

## Slide 2
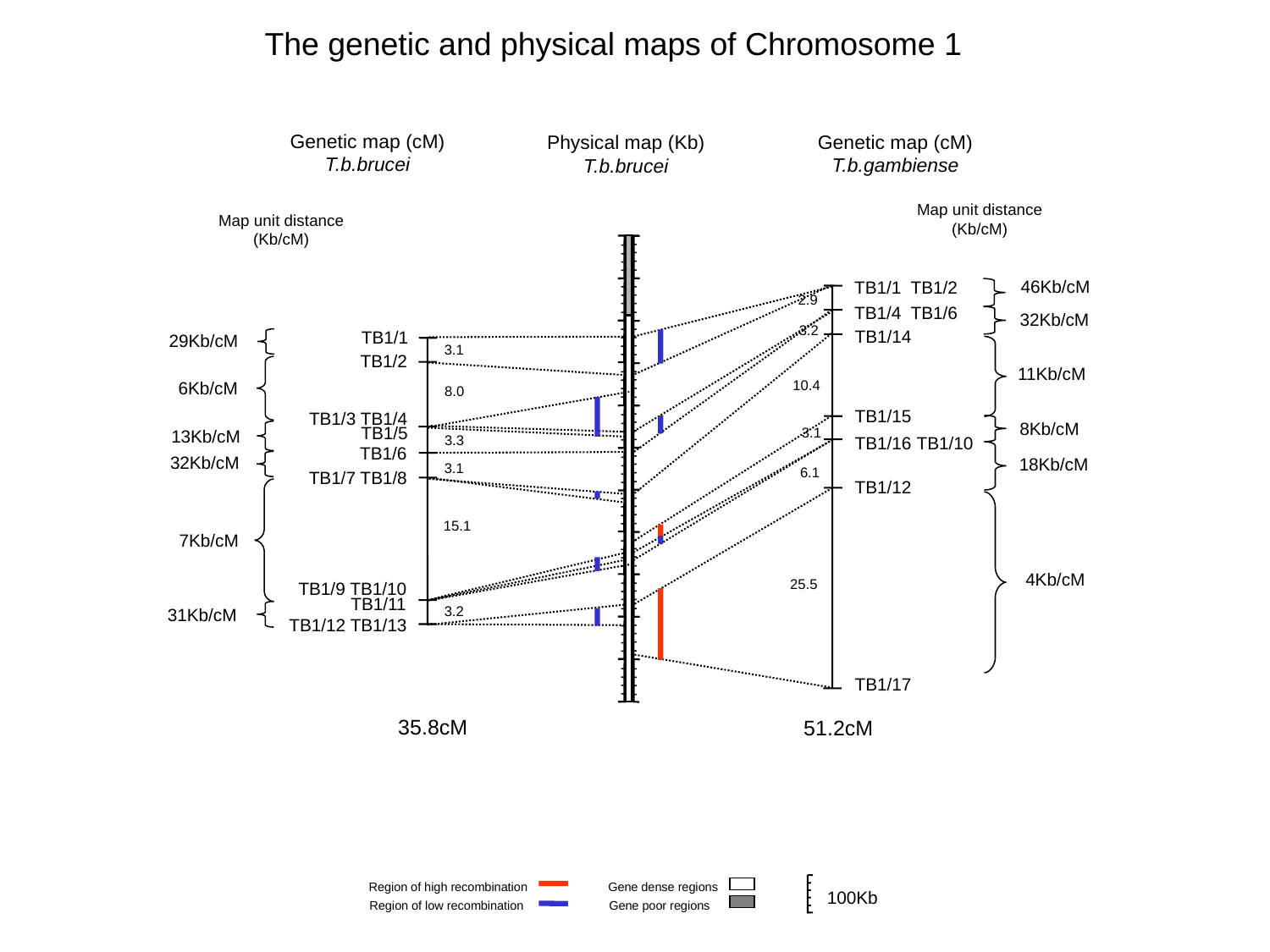

The genetic and physical maps of Chromosome 1
Genetic map (cM)
T.b.brucei
Genetic map (cM)
T.b.gambiense
Physical map (Kb)
T.b.brucei
Map unit distance (Kb/cM)
Map unit distance (Kb/cM)
46Kb/cM
TB1/1 TB1/2
2.9
TB1/4 TB1/6
32Kb/cM
3.2
TB1/14
TB1/1
29Kb/cM
3.1
TB1/2
11Kb/cM
10.4
6Kb/cM
8.0
TB1/15
 TB1/3 TB1/4
8Kb/cM
TB1/5
3.1
13Kb/cM
TB1/10
3.3
TB1/16
TB1/6
32Kb/cM
18Kb/cM
3.1
6.1
TB1/7 TB1/8
TB1/12
15.1
7Kb/cM
4Kb/cM
25.5
TB1/9 TB1/10
 TB1/11
3.2
31Kb/cM
 TB1/12 TB1/13
TB1/17
35.8cM
51.2cM
Region of high recombination
Gene dense regions
Gene poor regions
Region of low recombination
100Kb

## Slide 3
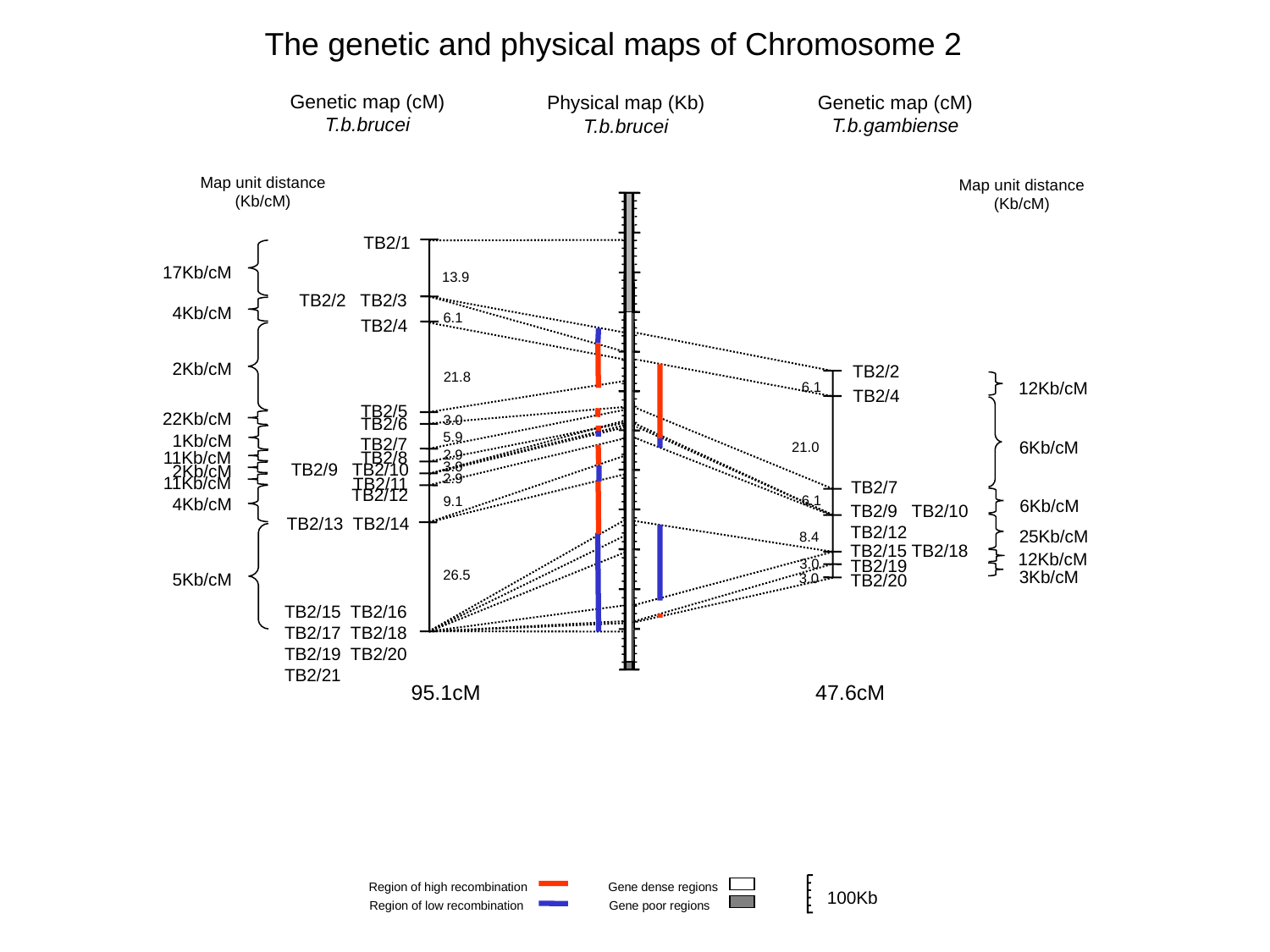

The genetic and physical maps of Chromosome 2
Genetic map (cM)
T.b.brucei
Genetic map (cM)
T.b.gambiense
Physical map (Kb)
T.b.brucei
Map unit distance (Kb/cM)
Map unit distance (Kb/cM)
TB2/1
17Kb/cM
13.9
TB2/2 TB2/3
4Kb/cM
6.1
TB2/4
2Kb/cM
TB2/2
21.8
12Kb/cM
6.1
TB2/4
TB2/5
22Kb/cM
3.0
TB2/6
5.9
1Kb/cM
TB2/7
6Kb/cM
21.0
2.9
11Kb/cM
TB2/8
3.0
2Kb/cM
2.9
TB2/9 TB2/10
11Kb/cM
TB2/11
TB2/7
TB2/12
6.1
9.1
4Kb/cM
6Kb/cM
TB2/9 TB2/10
TB2/12
TB2/13 TB2/14
25Kb/cM
8.4
TB2/15 TB2/18
12Kb/cM
3.0
TB2/19
3Kb/cM
26.5
5Kb/cM
TB2/20
3.0
TB2/15 TB2/16
TB2/17 TB2/18
TB2/19 TB2/20
TB2/21
95.1cM
47.6cM
Region of high recombination
Gene dense regions
Gene poor regions
Region of low recombination
100Kb

## Slide 4
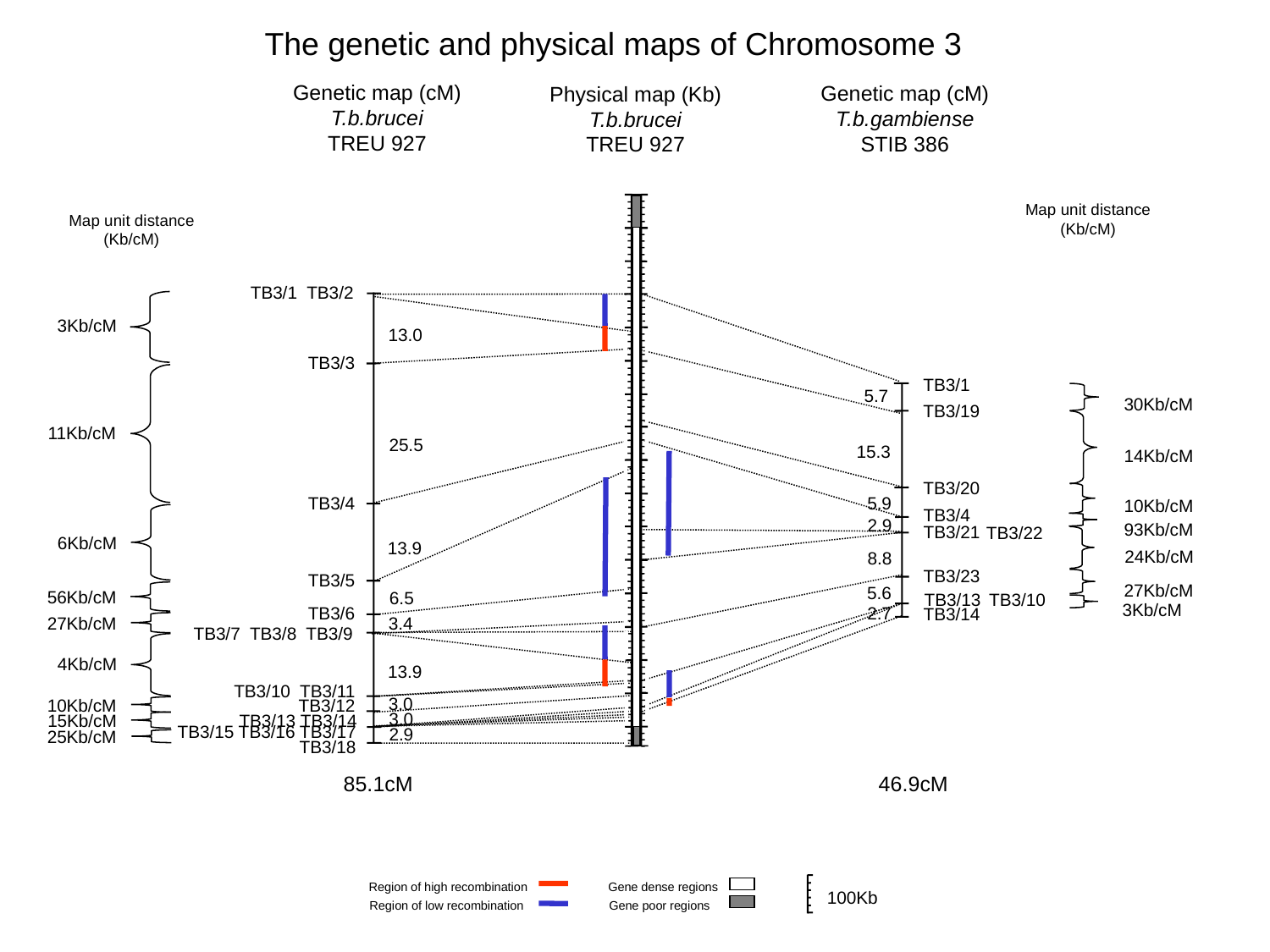

The genetic and physical maps of Chromosome 3
Genetic map (cM)
T.b.brucei
TREU 927
Genetic map (cM)
T.b.gambiense
STIB 386
Physical map (Kb)
T.b.brucei
TREU 927
Map unit distance (Kb/cM)
Map unit distance (Kb/cM)
TB3/1 TB3/2
3Kb/cM
13.0
TB3/3
TB3/1
30Kb/cM
14Kb/cM
10Kb/cM
93Kb/cM
24Kb/cM
27Kb/cM
3Kb/cM
5.7
TB3/19
11Kb/cM
25.5
15.3
TB3/20
TB3/4
5.9
TB3/4
2.9
TB3/21
TB3/22
6Kb/cM
13.9
8.8
TB3/5
TB3/23
56Kb/cM
6.5
5.6
TB3/13
TB3/10
TB3/6
2.7
TB3/14
27Kb/cM
3.4
TB3/7 TB3/8 TB3/9
4Kb/cM
13.9
TB3/10 TB3/11
3.0
10Kb/cM
TB3/12
3.0
15Kb/cM
TB3/13 TB3/14
TB3/15 TB3/16 TB3/17
2.9
25Kb/cM
TB3/18
85.1cM
46.9cM
Region of high recombination
Gene dense regions
Gene poor regions
Region of low recombination
100Kb

## Slide 5
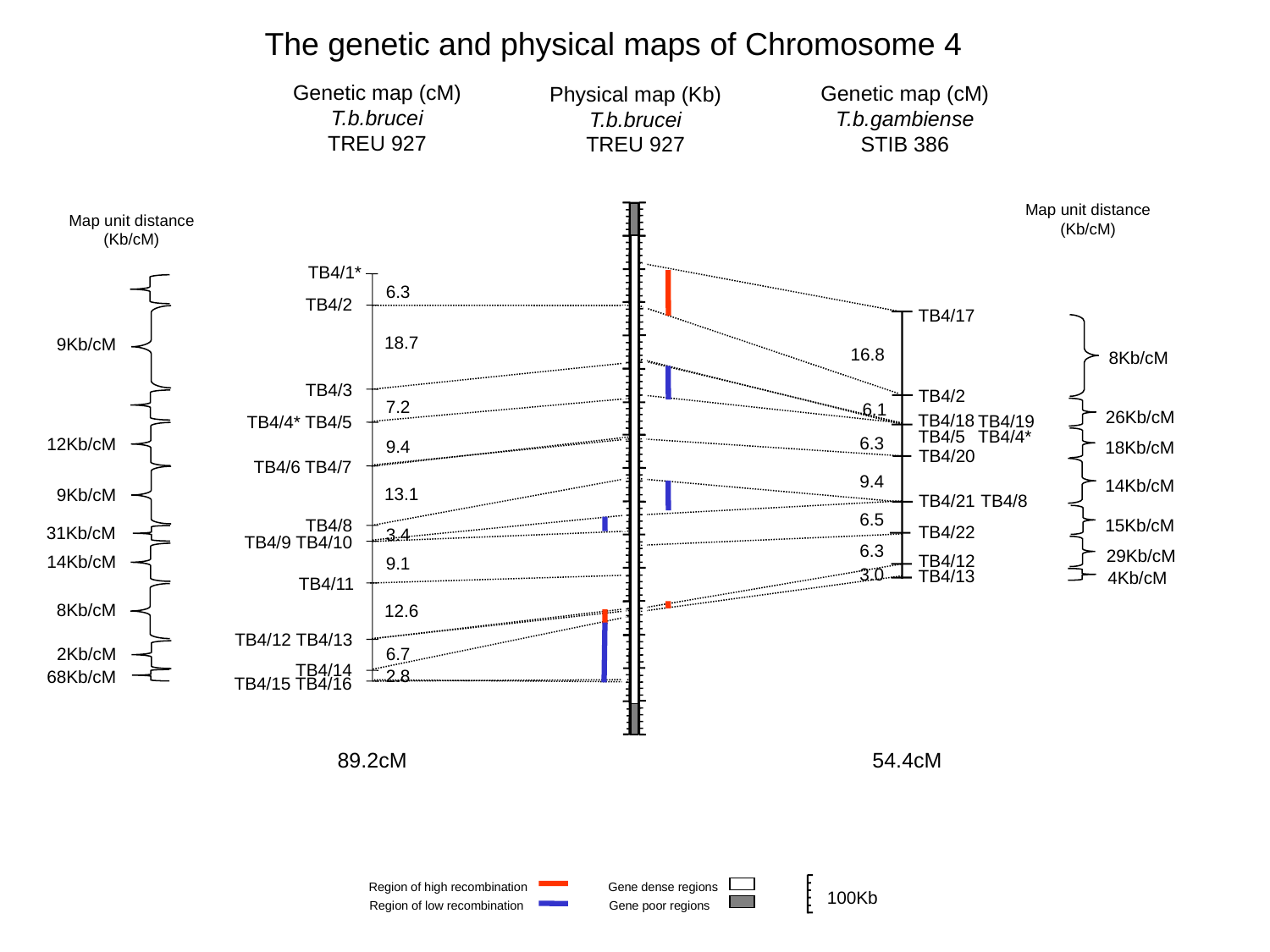

The genetic and physical maps of Chromosome 4
Genetic map (cM)
T.b.brucei
TREU 927
Genetic map (cM)
T.b.gambiense
STIB 386
Physical map (Kb)
T.b.brucei
TREU 927
Map unit distance (Kb/cM)
Map unit distance (Kb/cM)
TB4/1*
6.3
TB4/2
TB4/17
8Kb/cM
26Kb/cM
18Kb/cM
14Kb/cM
15Kb/cM
29Kb/cM
4Kb/cM
18.7
9Kb/cM
16.8
TB4/3
TB4/2
7.2
6.1
TB4/4* TB4/5
TB4/18
TB4/5
TB4/19
TB4/4*
12Kb/cM
9.4
6.3
TB4/20
TB4/6 TB4/7
9.4
9Kb/cM
13.1
TB4/21
TB4/8
TB4/8
6.5
31Kb/cM
3.4
TB4/22
TB4/9 TB4/10
6.3
14Kb/cM
9.1
TB4/12
3.0
TB4/13
TB4/11
8Kb/cM
12.6
TB4/12 TB4/13
6.7
2Kb/cM
TB4/14
2.8
68Kb/cM
TB4/15 TB4/16
89.2cM
54.4cM
Region of high recombination
Gene dense regions
Gene poor regions
Region of low recombination
100Kb

## Slide 6
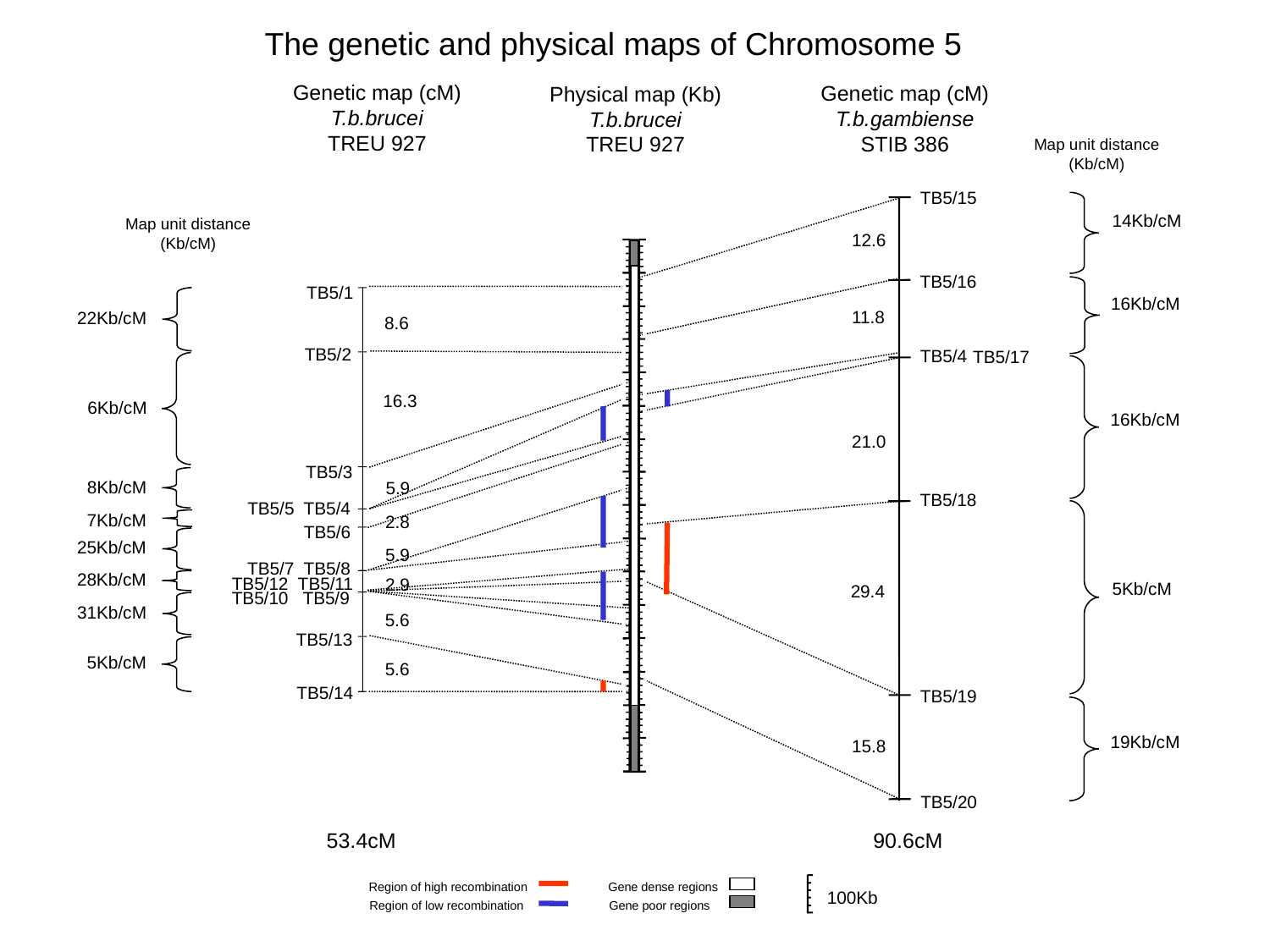

The genetic and physical maps of Chromosome 5
Genetic map (cM)
T.b.brucei
TREU 927
Genetic map (cM)
T.b.gambiense
STIB 386
Physical map (Kb)
T.b.brucei
TREU 927
Map unit distance (Kb/cM)
TB5/15
14Kb/cM
16Kb/cM
16Kb/cM
5Kb/cM
19Kb/cM
Map unit distance (Kb/cM)
12.6
TB5/16
TB5/1
22Kb/cM
8.6
11.8
TB5/2
TB5/4
TB5/17
16.3
6Kb/cM
21.0
TB5/3
8Kb/cM
5.9
TB5/18
TB5/5 TB5/4
7Kb/cM
2.8
TB5/6
25Kb/cM
5.9
TB5/7 TB5/8
28Kb/cM
2.9
TB5/12 TB5/11
TB5/10 TB5/9
29.4
31Kb/cM
5.6
TB5/13
5Kb/cM
5.6
TB5/14
TB5/19
15.8
TB5/20
90.6cM
53.4cM
Region of high recombination
Gene dense regions
Gene poor regions
Region of low recombination
100Kb

## Slide 7
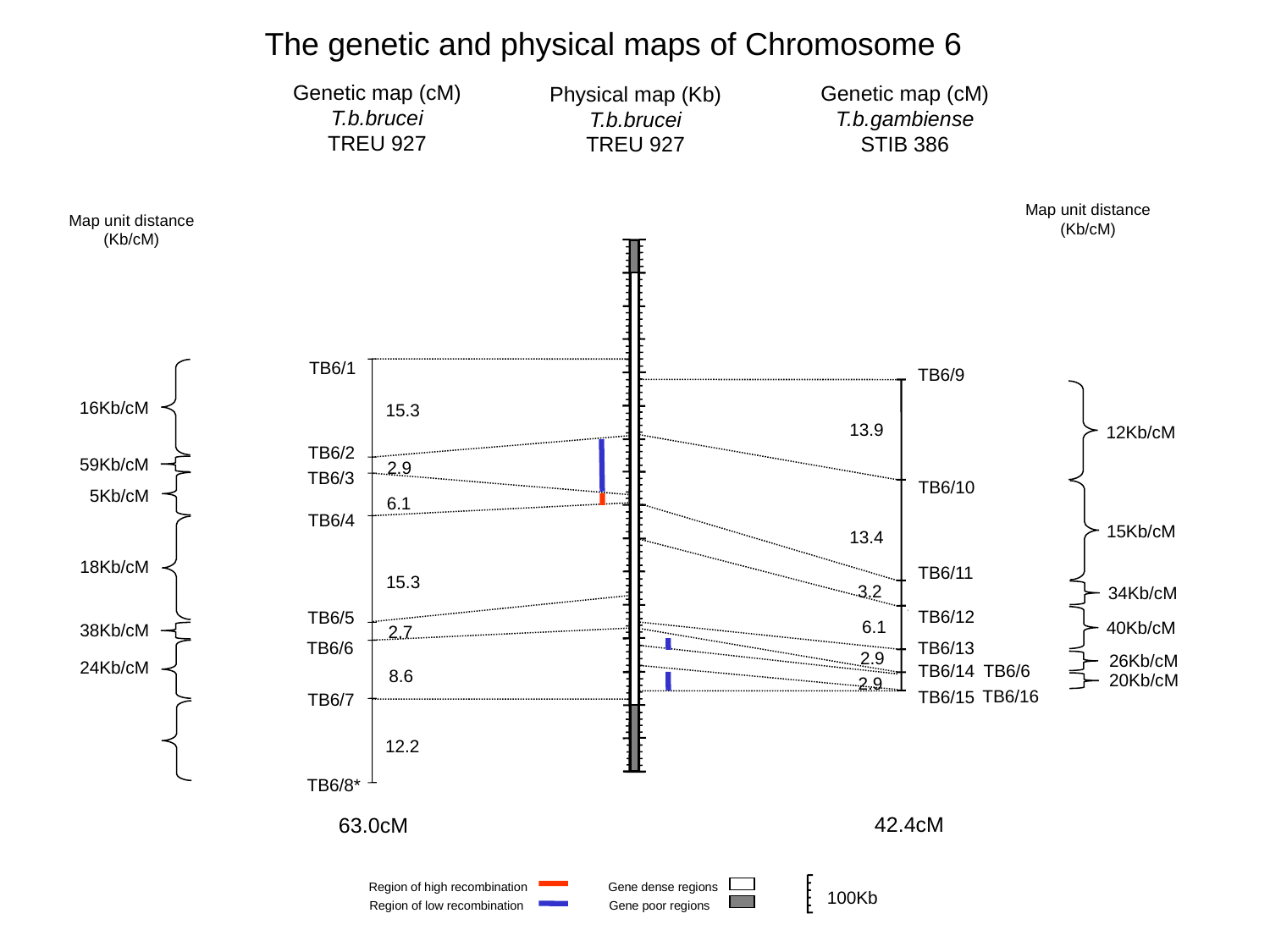

The genetic and physical maps of Chromosome 6
Genetic map (cM)
T.b.brucei
TREU 927
Genetic map (cM)
T.b.gambiense
STIB 386
Physical map (Kb)
T.b.brucei
TREU 927
Map unit distance (Kb/cM)
Map unit distance (Kb/cM)
TB6/1
TB6/9
16Kb/cM
15.3
12Kb/cM
13.9
TB6/2
59Kb/cM
2.9
TB6/3
TB6/10
5Kb/cM
6.1
TB6/4
15Kb/cM
13.4
18Kb/cM
TB6/11
15.3
34Kb/cM
3.2
TB6/5
TB6/12
40Kb/cM
38Kb/cM
2.7
6.1
TB6/6
TB6/13
26Kb/cM
2.9
24Kb/cM
8.6
TB6/6
TB6/14
20Kb/cM
2.9
TB6/16
TB6/7
TB6/15
12.2
TB6/8*
42.4cM
63.0cM
Region of high recombination
Gene dense regions
Gene poor regions
Region of low recombination
100Kb

## Slide 8
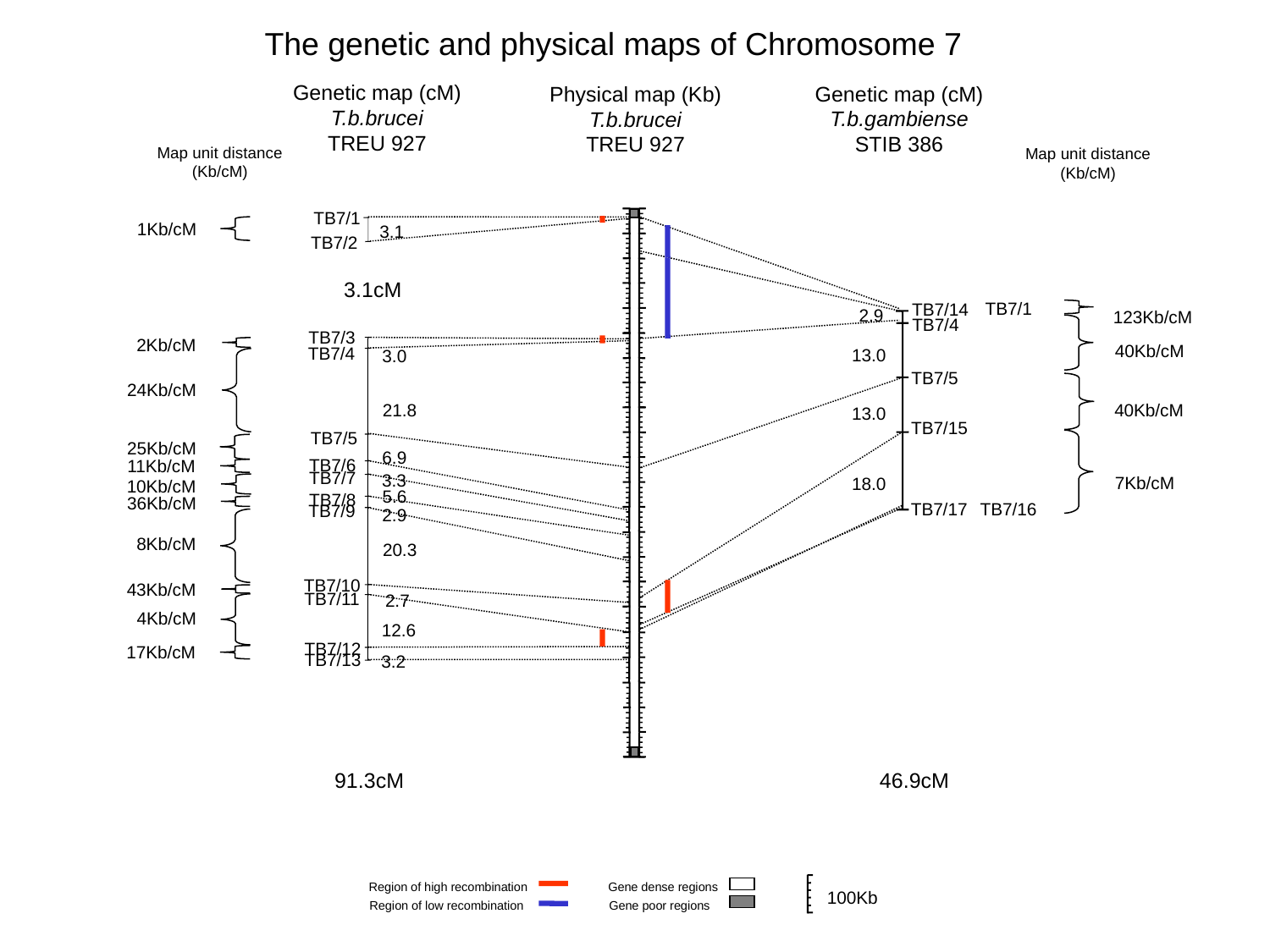

The genetic and physical maps of Chromosome 7
Genetic map (cM)
T.b.brucei
TREU 927
Physical map (Kb)
T.b.brucei
TREU 927
Genetic map (cM)
T.b.gambiense
STIB 386
Map unit distance (Kb/cM)
Map unit distance (Kb/cM)
TB7/1
1Kb/cM
3.1
TB7/2
3.1cM
TB7/1
TB7/14
123Kb/cM
40Kb/cM
40Kb/cM
7Kb/cM
2.9
TB7/4
TB7/3
2Kb/cM
TB7/4
3.0
13.0
TB7/5
24Kb/cM
21.8
13.0
TB7/15
TB7/5
25Kb/cM
6.9
TB7/6
11Kb/cM
TB7/7
3.3
10Kb/cM
18.0
5.6
TB7/8
36Kb/cM
TB7/9
2.9
TB7/17
TB7/16
8Kb/cM
20.3
TB7/10
43Kb/cM
TB7/11
2.7
4Kb/cM
12.6
TB7/12
17Kb/cM
TB7/13
3.2
46.9cM
91.3cM
Region of high recombination
Gene dense regions
Gene poor regions
Region of low recombination
100Kb

## Slide 9
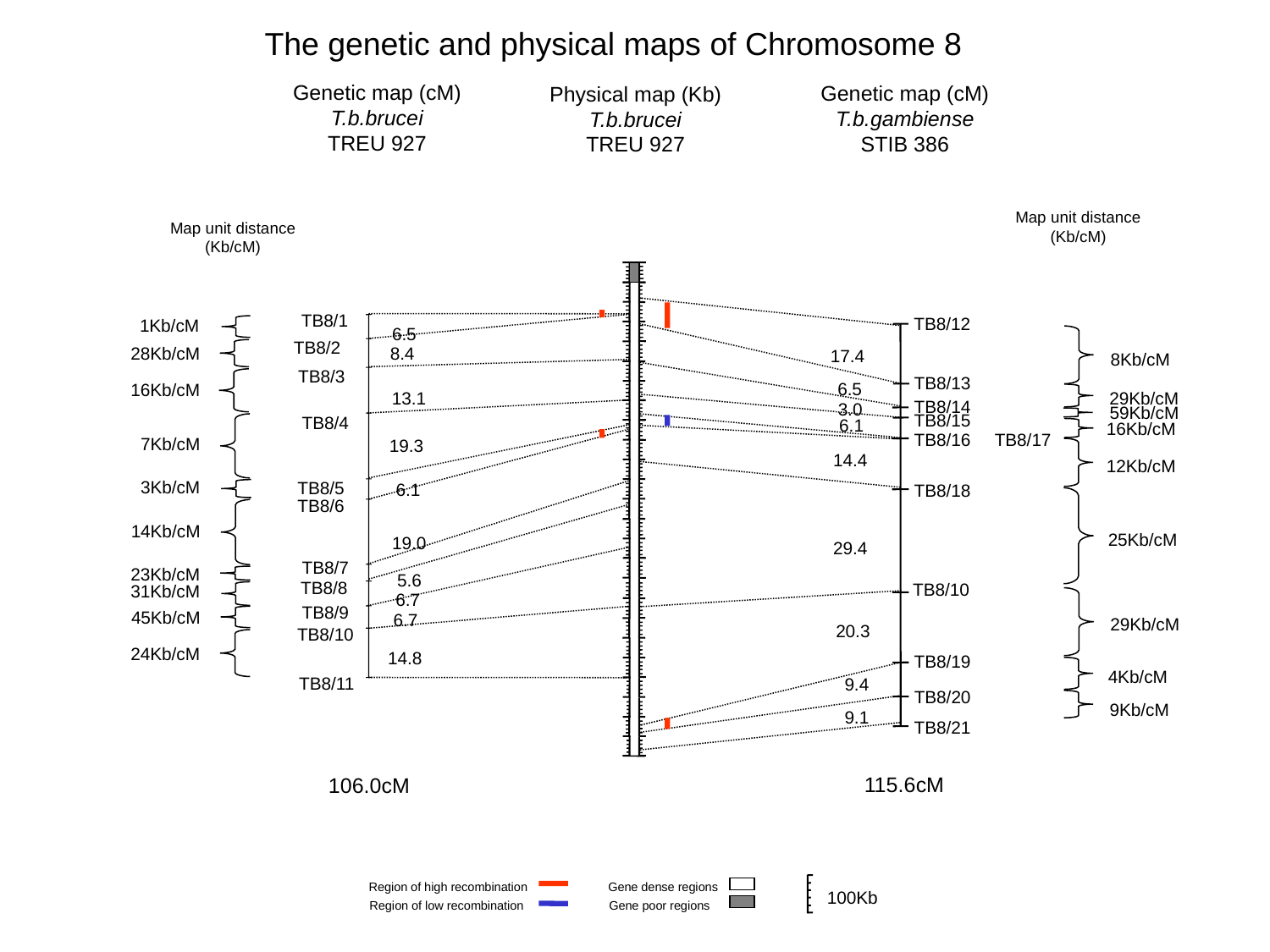

The genetic and physical maps of Chromosome 8
Genetic map (cM)
T.b.brucei
TREU 927
Genetic map (cM)
T.b.gambiense
STIB 386
Physical map (Kb)
T.b.brucei
TREU 927
Map unit distance (Kb/cM)
Map unit distance (Kb/cM)
TB8/1
TB8/12
1Kb/cM
6.5
8Kb/cM
29Kb/cM
59Kb/cM
16Kb/cM
12Kb/cM
25Kb/cM
29Kb/cM
4Kb/cM
9Kb/cM
TB8/2
28Kb/cM
8.4
17.4
TB8/3
TB8/13
6.5
16Kb/cM
13.1
TB8/14
3.0
TB8/15
TB8/4
6.1
TB8/16 TB8/17
7Kb/cM
19.3
14.4
3Kb/cM
TB8/5
6.1
TB8/18
TB8/6
14Kb/cM
19.0
29.4
TB8/7
23Kb/cM
5.6
TB8/8
TB8/10
31Kb/cM
6.7
TB8/9
45Kb/cM
6.7
20.3
TB8/10
24Kb/cM
14.8
TB8/19
TB8/11
9.4
TB8/20
9.1
TB8/21
115.6cM
106.0cM
Region of high recombination
Gene dense regions
Gene poor regions
Region of low recombination
100Kb

## Slide 10
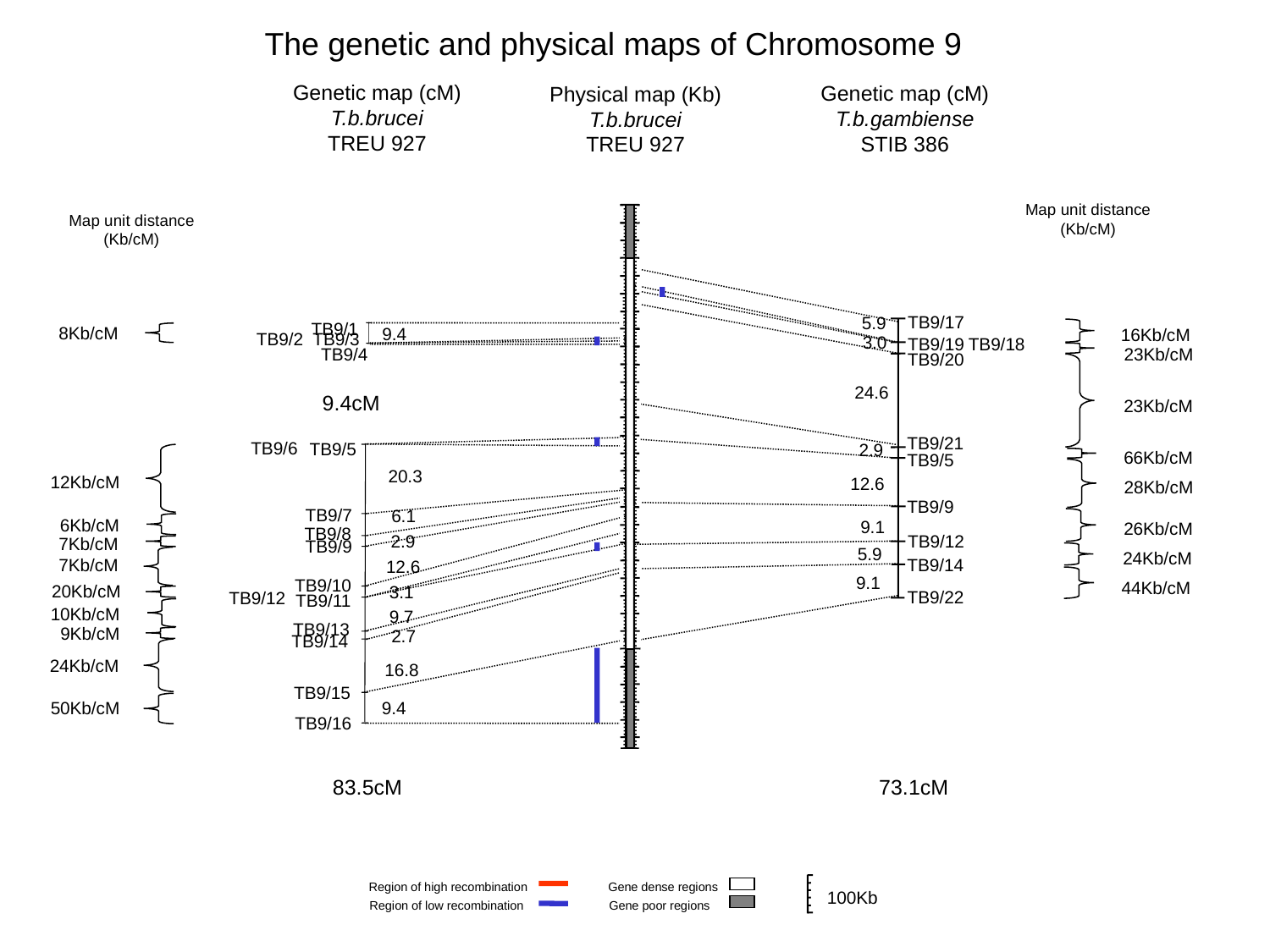

The genetic and physical maps of Chromosome 9
Genetic map (cM)
T.b.brucei
TREU 927
Genetic map (cM)
T.b.gambiense
STIB 386
Physical map (Kb)
T.b.brucei
TREU 927
Map unit distance (Kb/cM)
Map unit distance (Kb/cM)
TB9/17
TB9/1
5.9
8Kb/cM
9.4
16Kb/cM
23Kb/cM
23Kb/cM
66Kb/cM
28Kb/cM
26Kb/cM
24Kb/cM
44Kb/cM
TB9/2 TB9/3
 TB9/4
3.0
TB9/19
TB9/18
TB9/20
24.6
9.4cM
TB9/6
TB9/5
20.3
TB9/7
6.1
TB9/8
2.9
TB9/9
12.6
TB9/10
3.1
TB9/12
TB9/11
9.7
TB9/13
2.7
TB9/14
16.8
TB9/15
9.4
TB9/16
TB9/21
2.9
TB9/5
12Kb/cM
12.6
TB9/9
6Kb/cM
9.1
7Kb/cM
TB9/12
5.9
7Kb/cM
TB9/14
9.1
20Kb/cM
TB9/22
10Kb/cM
9Kb/cM
24Kb/cM
50Kb/cM
83.5cM
73.1cM
Region of high recombination
Gene dense regions
Gene poor regions
Region of low recombination
100Kb

## Slide 11
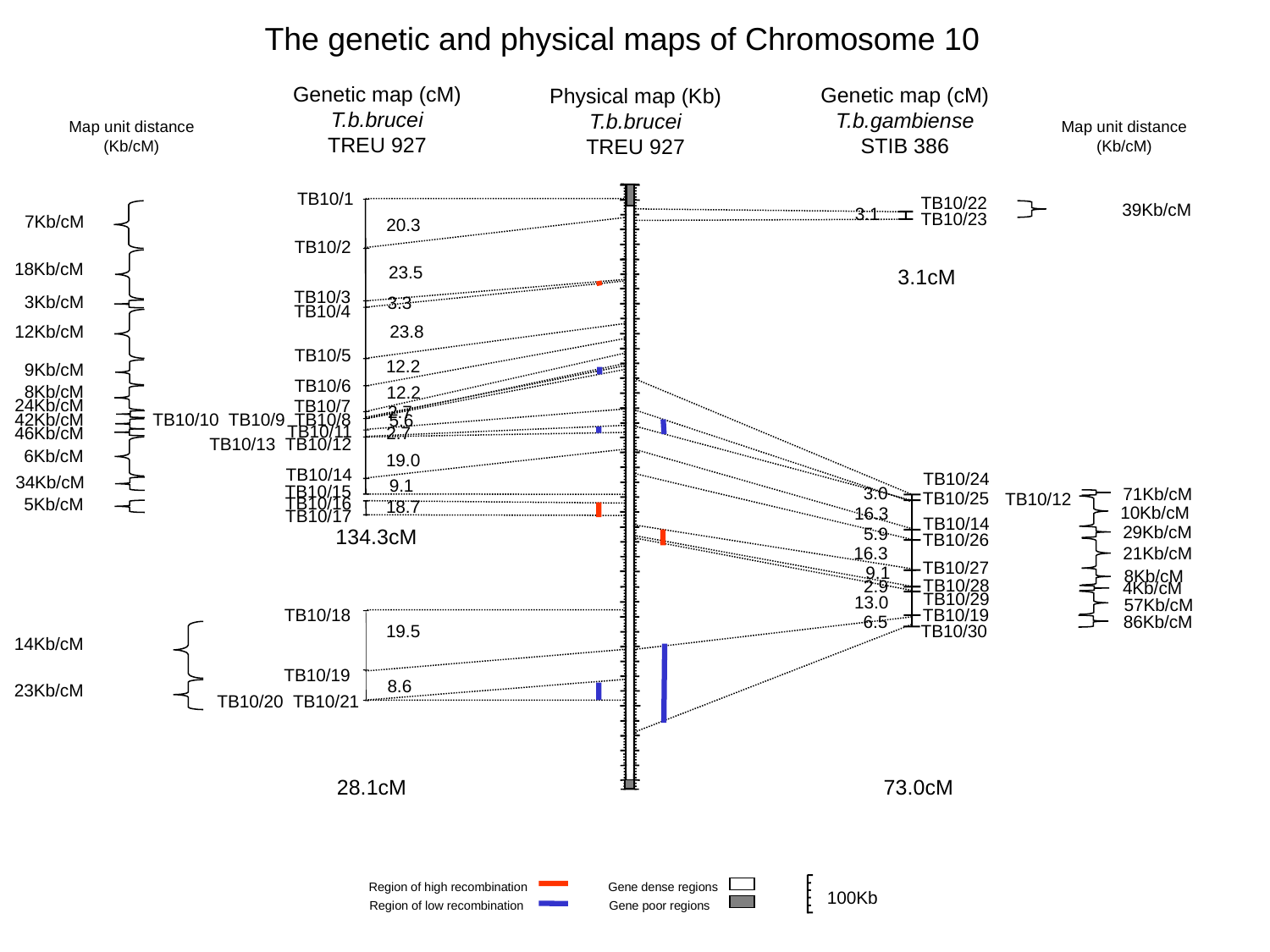

The genetic and physical maps of Chromosome 10
Genetic map (cM)
T.b.brucei
TREU 927
Genetic map (cM)
T.b.gambiense
STIB 386
Physical map (Kb)
T.b.brucei
TREU 927
Map unit distance (Kb/cM)
Map unit distance (Kb/cM)
TB10/1
TB10/22
39Kb/cM
3.1
7Kb/cM
20.3
TB10/23
TB10/2
18Kb/cM
23.5
3.1cM
TB10/3
3Kb/cM
3.3
TB10/4
12Kb/cM
23.8
TB10/5
12.2
9Kb/cM
TB10/6
8Kb/cM
12.2
24Kb/cM
TB10/7
2.7
42Kb/cM
TB10/10 TB10/9 TB10/8
5.6
TB10/11
46Kb/cM
2.7
TB10/13 TB10/12
6Kb/cM
19.0
TB10/14
34Kb/cM
TB10/24
9.1
TB10/15
71Kb/cM
3.0
5Kb/cM
TB10/16
TB10/25
TB10/12
18.7
10Kb/cM
TB10/17
16.3
TB10/14
29Kb/cM
134.3cM
5.9
TB10/26
21Kb/cM
16.3
TB10/27
8Kb/cM
9.1
4Kb/cM
TB10/28
2.9
57Kb/cM
TB10/29
13.0
TB10/18
TB10/19
86Kb/cM
6.5
19.5
TB10/30
14Kb/cM
TB10/19
8.6
23Kb/cM
TB10/20 TB10/21
28.1cM
73.0cM
Region of high recombination
Gene dense regions
Gene poor regions
Region of low recombination
100Kb

## Slide 12
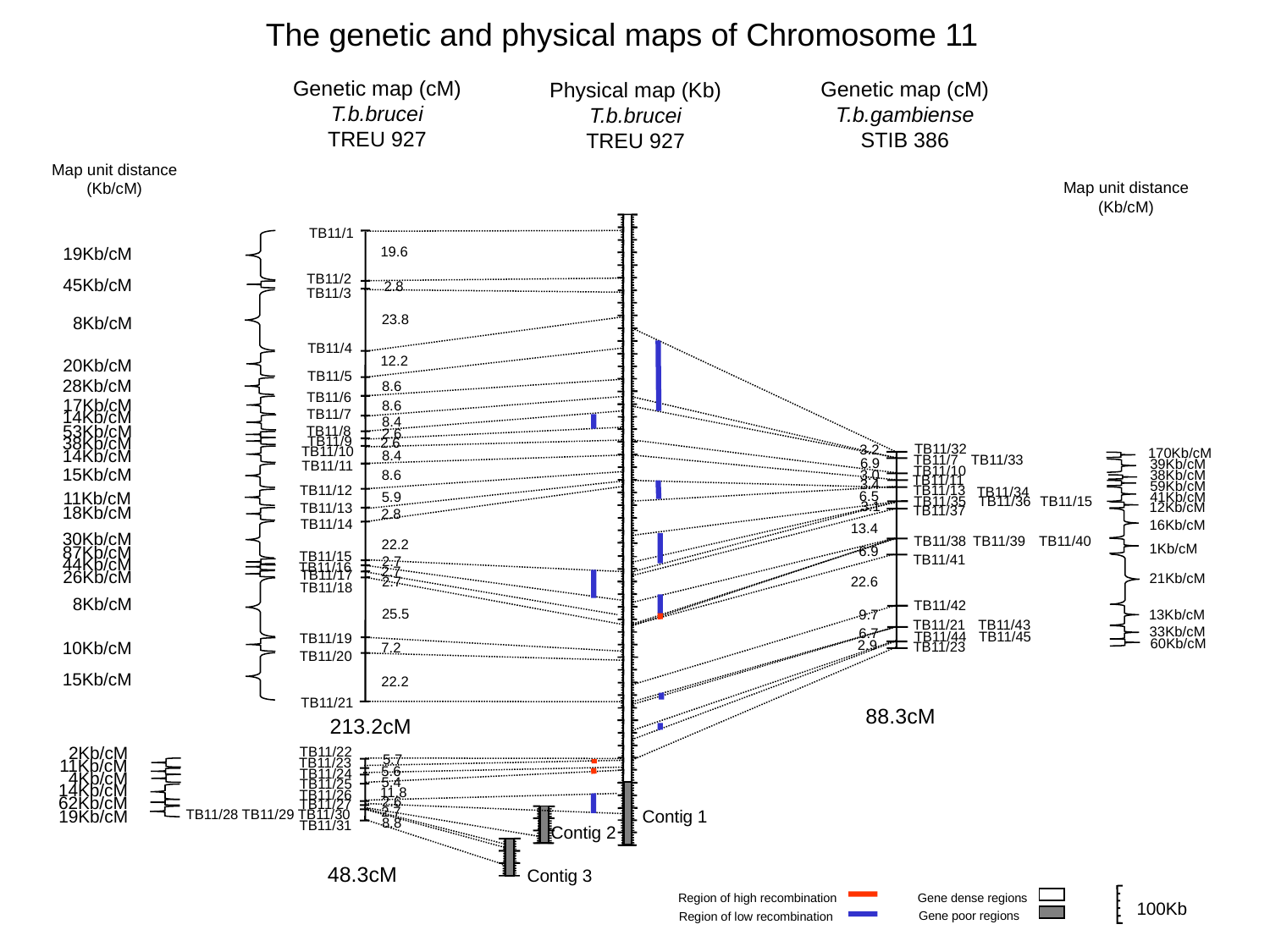

The genetic and physical maps of Chromosome 11
Genetic map (cM)
T.b.brucei
TREU 927
Genetic map (cM)
T.b.gambiense
STIB 386
Physical map (Kb)
T.b.brucei
TREU 927
Map unit distance (Kb/cM)
Map unit distance (Kb/cM)
TB11/1
19Kb/cM
19.6
TB11/2
45Kb/cM
2.8
TB11/3
23.8
8Kb/cM
TB11/4
12.2
20Kb/cM
TB11/5
28Kb/cM
8.6
TB11/6
17Kb/cM
8.6
14Kb/cM
TB11/7
8.4
53Kb/cM
TB11/8
2.6
38Kb/cM
TB11/9
2.6
TB11/10
170Kb/cM
14Kb/cM
TB11/32
8.4
3.2
39Kb/cM
TB11/11
TB11/7
TB11/33
6.9
15Kb/cM
8.6
38Kb/cM
TB11/10
3.0
59Kb/cM
TB11/11
TB11/12
3.4
11Kb/cM
TB11/13
41Kb/cM
5.9
TB11/34
6.5
12Kb/cM
TB11/35
TB11/36
TB11/15
TB11/13
18Kb/cM
3.1
2.8
TB11/37
16Kb/cM
TB11/14
13.4
30Kb/cM
22.2
TB11/38
TB11/39
TB11/40
1Kb/cM
87Kb/cM
TB11/15
6.9
2.7
44Kb/cM
TB11/41
TB11/16
2.7
26Kb/cM
TB11/17
21Kb/cM
2.7
TB11/18
22.6
8Kb/cM
TB11/42
25.5
13Kb/cM
9.7
TB11/21
TB11/43
33Kb/cM
TB11/19
6.7
TB11/44
TB11/45
60Kb/cM
10Kb/cM
7.2
2.9
TB11/23
TB11/20
15Kb/cM
22.2
TB11/21
88.3cM
213.2cM
2Kb/cM
TB11/22
5.7
11Kb/cM
TB11/23
5.6
TB11/24
4Kb/cM
5.4
TB11/25
14Kb/cM
11.8
TB11/26
62Kb/cM
2.6
TB11/27
2.7
19Kb/cM
Contig 1
TB11/28 TB11/29 TB11/30
8.8
TB11/31
Contig 2
48.3cM
Contig 3
Region of high recombination
Gene dense regions
Gene poor regions
Region of low recombination
100Kb
