# Supplementary material for: Genetic analysis of the human infective trypanosome Trypanosoma brucei gambiense: chromosomal segregation, crossing over, and the construction of a genetic map
Source: Genome Biol. 2008 Jun 22;9(6):R103. doi: 10.1186/gb-2008-9-6-r103 (PMC2481433; doi:10.1186/gb-2008-9-6-r103)
Supplement: Additional data file 4 — The name and relevant genotypes of the parental strains and 38 unique F1 progeny derived from the STIB 386 × STIB 247 crosses that were analysed for the construction of the T. b. gambiense linkage map. Inheritance of marker alleles from both parents for 2 microsatellites (JS2 and PLC) and 3 minisatellites (CRAM, 292 and MS42) were used as genotyping markers. [file gb-2008-9-6-r103-S4.doc]

## Additional data file 4 – Unique progeny genotype data

| **Hybrids and Parents** | **Genotyping marker scores** | | | | |
| --- | --- | --- | --- | --- | --- |
| Identification name | JS2 | **PLC** | **MS42** | **292** | **CRAM** |
| **STIB 247 parental stock** | **5-6** | **5-5** | **5-5** | **5-5** | **1-1** |
| **STIB 386 parental stock** | **1-2** | **1-2** | **1-2** | **1-2** | **1-2** |
| F9/45 mcl 2 | 1-5 | 1-5 | 2-5 | 1-5 | 1-2 |
| F9/45 mcl 10 | 1-6 | 2-5 | 1-5 | 2-5 | 1-1 |
| F9/45 mcl 11 | 1-5 | 2-5 | 1-5 | 1-5 | 1-2 |
| F9/45 mcl 12 | 1-5 | 2-5 | - | 2-5 | 1-1 |
| F9/34 mcl 1 | 2-5 | 1-5 | 1-5 | 2-5 | 1-1 |
| B80 cl 2 | 1-5 | 2-5 | 2-5 | 2-5 | 1-2 |
| F492/50 bscl 1 | 2-6 | 2-5 | 1-5 | 2-5 | 1-1 |
| F492/50 bscl 6 | 1-5 | 1-5 | - | 1-5 | 1-2 |
| F492/50 bscl 8 | 1-6 | 2-5 | 1-5 | 2-5 | 1-1 |
| F492/50 bscl 9 | 2-5 | 2-5 | 2-5 | 1-5 | 1-2 |
| F492/50 bscl 12 | 2-5 | 1-5 | 2-5 | 2-5 | 1-1 |
| F492/50 bscl 14 | 1-6 | 1-5 | 1-5 | 1-5 | 1-1 |
| F492/50 bscl 21 | 2-6 | 2-5 | 1-5 | 1-5 | 1-1 |
| F492/50 bscl 23 | 2-5 | 2-5 | 1-5 | 1-5 | 1-2 |
| F492/50 bscl 5/1b | 2-6 | 2-5 | 1-5 | 1-5 | 1-2 |
| F9/41 bscl 5 | 1-5 | 2-5 | 2-5 | 2-5 | 1-2 |
| F9/41 bscl 7 | 1-5 | 1-5 | 2-5 | 2-5 | 1-2 |
| F9/41 bscl 9 | 2-6 | 2-5 | 1-5 | 1-5 | 1-1 |
| F29/46 bscl 3 | 2-6 | 2-5 | 1-5 | 1-5 | 1-1 |
| F29/46 bscl 4 | 2-6 | 1-5 | 1-5 | 1-5 | 1-1 |
| F19/31clone 1 | 1-6 | 1-5 | 1-5 | 2-5 | 1-2 |
| F 19/31 bscl 11 | 1-5 | 2-5 | 1-5 | 1-5 | 1-1 |
| F28/46 bscl 6 | 2-5 | 2-5 | 1-5 | 1-5 | 1-2 |
| F28/46 bscl 11 | 2-5 | 2-5 | 2-5 | 2-5 | 1-2 |
| F29/46 bscl 2 | 2-6 | 2-5 | 1-5 | 1-5 | 1-1 |
| F28/46 bscl 1 | 2-5 | 1-5 | 1-5 | 1-5 | 1-1 |
| F28/46 bscl 4 | 2-6 | 2-5 | 1-5 | 1-5 | 1-2 |
| F28/46 bscl 7 | 2-5 | 1-5 | 1-5 | 2-5 | 1-1 |
| F28/46 bscl 8 | 2-6 | 2-5 | 2-5 | 1-5 | 1-2 |
| F29/46 bscl 1 | 1-5 | 1-5 | 1-5 | 2-5 | 1-1 |
| F9/41 bscl 1 | 1-6 | 1-5 | 2-5 | 2-5 | 1-1 |
| F9/41 bscl 2 | 1-6 | 1-5 | 1-5 | 1-5 | 1-1 |
| F9/41 bscl 8 | 1-5 | 1-5 | 1-5 | 2-5 | 1-1 |
| F9/41 bscl 11 | 1-5 | 2-5 | 1-5 | 1-5 | 1-2 |
| F19/31 bscl 5 | 1-6 | 2-5 | 1-5 | 1-5 | 1-1 |
| F19/31 bscl 10 | 2-6 | 2-5 | 1-5 | 1-5 | 1-2 |
| F19/31 bscl 8 | 1-6 | 2-5 | 1-5 | 1-5 | 1-2 |
| F492/50 bscl 7 | 2-5 | 2-5 | 1-5 | 1-5 | 1-2 |

The name and relevant genotypes of the parental strains and 38 unique F1 progeny derived from the STIB 386 X STIB 247 crosses that were analysed for the construction of the *T.b.gambiense* linkage map. Inheritance of marker alleles from both parents for 2 microsatellites (JS2 and PLC) and 3 minisatellites (CRAM, 292 and MS42) were used as genotyping markers.
